# PCR-based CRISPR/Cas9 system for fluorescent tagging: A tool for studying *Candida parapsilosis* virulence

**DOI:** 10.1371/journal.pone.0312948

**Published:** 2025-02-24

**Authors:** Tibor Nemeth, Andrea Zarnocki, Anett Ladanyi, Csaba Papp, Ferhan Ayaydin, Gabor Janos Szebeni, Attila Gacser

**Affiliations:** 1 Department of Biotechnology and Microbiology, Faculty of Science and Informatics, University of Szeged, Szeged, Hungary; 2 Functional Cell Biology and Immunology Advanced Core Facility (FCBI-ACF), Hungarian Centre of Excellence for Molecular Medicine (HCEMM), University of Szeged, Szeged, Hungary; 3 Agribiotechnology and Precision Breeding for Food Security National Laboratory, Institute of Plant Biology, HUN-REN Biological Research Centre, Szeged, Hungary; 4 Laboratory of Functional Genomics, Core Facility, HUN-REN Biological Research Centre, Szeged, Hungary; 5 Department of Internal Medicine, Hematology Centre, Faculty of Medicine, University of Szeged, Szeged, Hungary; 6 HCEMM-SZTE Pathogen Fungi Research Group, University of Szeged, Szeged, Hungary; 7 HUN-REN-SZTE Pathomechanisms of Fungal Infections Research Group, University of Szeged, Szeged, Hungary; 8 IKIKK, Competence Centre for Molecular Biology, Bionics and Biotechnology, University of Szeged, Szeged, Hungary; Lebanese American University, LEBANON

## Abstract

*Candida parapsilosis* is persistent in a hospital environment hence it is often associated with nosocomial infections especially amongst low-birth weight neonates. Genetic modification is therefore important to characterise the physiological and virulence related properties of this fungus. A PCR-based CRISPR/Cas9 system has been adopted to facilitate the generation of fluorescent tagged prototroph isolates. We examined a total of eight fluorescent protein coding genes, out of which three were found to be applicable for simultaneous utilisation. We investigated three clinical isolates of *C. parapsilosis* in terms of their adherence to silicone and their uptake by J774.2 murine macrophages in competition assays. Interestingly, we found significant differences between them in both experiments where GA1 isolate was significantly less resistant to macrophage uptake and CDC317 was significantly more adherent to silicone material. *In silico* analysis of the agglutinin-like sequences (Als) exposed remarkable diversity in this protein family and additionally, the thorough analysis of the *ALS* genes revealed evidence of formation of a new gene by intrachromosomal recombination in the GA1 isolate. Finally, we provide a step by step protocol for the application of the PCR-based CRISPR/Cas9 system for fluorescently labelling *C. parapsilosis* isolates.

## Introduction

*Candida* species are responsible for serious life-threatening systemic infections affecting nearly one million immunocompromised individuals annually. However, the number of the known *Candida* species identified to date exceeds 200, only 6-7 account for the majority of the cases [1,2]. Amongst these, *Candida parapsilosis* primarily affects neonates with low birth weight [3]. This species shares several similarities with the predominant pathogen, *Candida albicans* with regard to its diploid nature and the alternative translation of the CUG codon to serine instead of leucine [4]. *C. parapsilosis* tends to colonise the skin, and can be isolated from the surface of medical devices and catheters as well as from the hand of healthcare workers which is considered the main route of infection in a hospital environment and source of outbreaks of infections in these institutions [5–7]. Investigation of the virulence properties of this fungus is therefore demanding. Early approaches for genome alteration in *C. parapsilosis* were either time consuming (FLP-FRT system) or required a special laboratory strain for double auxotrophy complementation and none of them enabled the generation of cumulative mutations. Additionally, there is always an exogenous sequence left behind at the site of the modification that might interfere with the biological function [8,9].

The introduction of the clustered regularly interspaced short palindromic repeats (CRISPR)/Cas9 approach revolutionised the nucleotide precise genome editing in various species. Since its first description as the adaptive immune system of prokaryotes against genome invading viral elements, it has been adopted with great success in typical eukaryotic models [10–17]. The functional Cas9 ribonucleoprotein consists of the Cas9 endonuclease and two RNA molecules called the CRISPR RNA and the trans-activating CRISPR RNA that however, turned out to be functional even if artificially joined and transcribed as one single guide RNA (sgRNA). The first twenty nucleotides at the 5’ end of the sgRNA define the site of the cleavage by sequence complementarity with the target DNA, but only if the target lies upstream of a short genomic sequence called the protospacer adjacent motif (PAM) [18,19]. Once the double strand break (DSB) is introduced, the repair mechanisms of the cell are activated to either ligate the free DNA ends (non-homologous end joining) or use the homologous chromosome as a template (homology directed repair). The former can lead to frameshift and often nonsense mutations, the latter is very likely to restore the original sequence. The recombination machinery can be influenced however, when an alternative template (repair template or donor DNA – dDNA) is present in the cell with sequences homologous to the affected region that can be exploited in targeted mutagenesis. Out of the described Cas9s to date, Type II Cas9s are amongst the most preferred, especially the one deriving from *Streptococcus pyogenes* (SpCas9) as it has the shortest PAM sequence (5’–NGG–3’) that maximises the number of the potential editing sites [20]. The first application of CRISPR/Cas9 for genome editing in *Candida* species dates back to 2015, when Vyas and co-workers developed a system for *C. albicans* with both the Cas9 and the sgRNA coding genes permanently integrated into the genome [21]. Soon Min and colleagues demonstrated that even transient expression of the Cas9 and sgRNA is sufficient for gene deletions in *C. albicans* [22]. A year later the problem of marker recycling has been solved permitting the generation of cumulative mutations [23]. Besides, a PCR-based approach has also been presented utilising an integrative construct that is assembled *in vivo* after co-transforming the fungus with two fragments. After genome editing, the construct can be excised from the genome by stochastic recombination or via a directed event catalysed by the site specific recombinase flippase (FLP) to enable the subsequent use of the system [24]. Over the last years the CRISPR/Cas9 method has been introduced in several other *Candida* species, including *C. glabrata*, *C. lusitaniae*, *C. tropicalis*, *C. auris* and the neonatal pathogen *C. parapsilosis* and its closely related sister species [25–30]. This latter application utilises an autonomously replicating plasmid carrying a nourseothricin (NTC) selectable marker. The integration of the sgRNA target sequence follows the idea of Ng and Dean where a pair of short oligonucleotides complementary to each other are annealed and ligated with the TypeIIS type restriction endonuclease, *Sap*I digested plasmid. The method was suitable to introduce STOP codons, delete and reintegrate genes in *C. parapsilosis sensu lato* species [29–31].

We have previously identified a locus in *C. parapsilosis* (CpNEUT5L) and developed a set of compatible plasmids for gene overexpression including GFP that permitted us to characterise the uptake of the yeast cells by murine macrophages. Using a fluorescent protein (FP) expressing strain not only facilitates the investigation by eliminating the necessity of labeling with fluorescent dyes but it also permits the observation of living cells for longer co-incubation periods [32,33]. This workflow enabled us to modify different isolates, however no other mutations could have been generated due to the lack of alternative selection markers, and only GFP ORF was available for overexpression.

Here we introduce a PCR-based CRISPR/Cas9 technique and a collection of FP coding plasmids for dDNA generation. The proper combination of FPs enabled us to follow three different isolates of this species for the first time in a competition assay. Besides corroborating the intraspecies heterogeneity indicated by former studies in terms of virulence related aspects in the human pathogenic fungus *C. parapsilosis*, we have found an evidence for the emergence of a new gene by recombination via repetitive sequences. Finally, we were able to characterise the performance of CRISPR/Cas9 technique in knock-in applications. In line with these findings we wish to highlight the importance of thorough mutant validation and make suggestions on the most adequate use of the CRISPR/Cas9 technique.

## Materials and methods

Strains used in this study are listed in S1 Table.

Oligonucleotides used in this study are listed in S2 Table.

### Growth conditions

*E. coli* 2T1 strain was cultivated in LB media (1% (m/V) NaCl, 1% (m/V) tryptone, 0.5% (m/V) yeast extract without antibiotics. For solid media 1.5% (m/V) agar was added. For selection, kanamycin (Merck) or ampicillin (Merck) was supplemented at a final concentration of 50 µg/ml.

*C. parapsilosis* isolates were stored in YPD medium (1% (m/V) glucose, 1% (m/V) peptone, 1% (m/V) yeast extract) containing 20% (V/V) glycerol at -80 °C. Strains were maintained on YPD solid medium supplemented with 1.5% (m/V) agar at 4 °C. For cultivation penicillin/streptomycin (Lonza) was applied in a final concentration of 100 unit/ml (YPD-PS). YPD-PS plates containing 2 or 100 µg/ml NTC (Jena Bioscience) were used upon transformation cassette excision and selection, respectively. YNB-Dropout-maltose (0.19 % (m/V) YNB, 2 % (m/V) maltose) media was applied for marker recycling. The strains were inoculated two days before the experiment in 2 ml of YPD-PS and were shaken (160 rpm) at 30 °C. On the next day, 0.3 µl suspension was pipetted into a new 2 ml YPD-PS media, and incubated under the same conditions for not more than 16 hours. For fluorescence imaging, the samples were protected from light. The cells were collected and washed twice with 1x PBS (2,800 g, 5 min), counted in a hemocytometer, and adjusted to the required concentration.

J774.2 macrophages suspended in cryoprotective medium (Basal Eagle’s Medium with Hanks’ balanced salt solution and 15% Dimethylsulfoxide without L-Glutamine) were stored in liquid nitrogen. Upon use, the cells were maintained in Dulbecco’s modified eagle medium supplemented with 10% (V/V) heat inactivated fetal bovine serum and 100 unit/ml penicillin/streptomycin solution (DMEM/FBS/PS) and stored at 37 °C, in the presence of 5% (V/V) CO_2_ and 100% (V/V) relative humidity. Macrophages were used for no more than six weeks (20 passages).

### Plasmid construction

Plasmids used in this study are listed in S3 Table.

#### p TB101.

To make the Cas9 plasmids compatible with *C. parapsilosis*, first the upstream target sequence for homologous recombination was modified from *C. albicans HIS1* to that of *C. parapsilosis*. A short sequence was amplified with the primer pair pADH99_fragment1_FOR - pADH99_fragment1_REV from the pADH99 and with pADH99_fragment2_FOR - pADH99_fragment2_REV from *C. parapsilosis* CLIB214 genomic DNA [24,34]. The fragments were isolated and stitched together by PCR using the primers pADH99_fragment1_FOR and pADH99_fragment2_REV. The amplicon and the pADH99 were digested with *Pvu*I/*Sal*I, and the plasmid backbone was ligated with the fragment to gain pADH99_1. Next, the maltose promoter was restored by amplifying this sequence from pADH99 with the primers Pmal_FOR and Pmal_REV. The amplicon was digested with *Xho*I, isolated and ligated with *Sal*I linearised pADH99_1 to create the pADH99_2 (*Sal*I and *Xho*I provide compatible ends). Proper orientation was checked by digestion. The regulator sequences of CPAR2_407690 (*CpTEF1*) were introduced as a fusion PCR product. The promoter and the terminator were amplified from *C. parapsilosis* CLIB214 genomic DNA with the primer pairs 407690Prom_FOR - 407690Prom_REV and 407690Ter_FOR - 407690Ter_REV respectively, isolated and fused by PCR using 407690Prom_FOR - 407690Ter_REV primer pair. The resulting amplicon (carrying recognition sites for *Age*I and *Nhe*I restriction endonucleases between the promoter and terminator, and flanked by incomplete recognition sites for *Sal*I and *KpnI*) was phosphorylated with polynucleotide kinase. The pADH99_2 was digested with *Kpn*I and the protruding 3’ was removed by Klenow fragment. The blunted plasmid backbone and the phosphorylated amplicon were ligated to obtain pADH99_3 resulting in the recovery of the *Kpn*I site at the terminator and introducing a *Sal*I recognition sequence at the promoter. Correct orientation was checked by digestion. Finally, to reintroduce the Cas9 coding gene, Cas9_AgeI_FOR and Cas9_NheI_REV primer pair was used to amplify the Cas9 from pCP-tRNA [29,30]. The amplicon and pADH99_3 were digested with the respective enzymes, isolated and ligated to create pTB101.

#### p TB120.

First, the *C. albicans* SNR52 promoter on pADH110 was replaced to that of *C. parapsilosis* [24]. The fragment 110-II was amplified from genomic DNA of *C. parapsilosis* CLIB214 with primers 110-II_FOR – 110-II_REV [34]. The fragment 110-I and fragment 110-III were amplified from the pADH110 with primer pairs AHO1096 - 110-I_REV and 110-III_FOR - 110-III_REV respectively. All three fragments were isolated and stitched together in a PCR using the primers AHO1096 and 110-III_REV. The amplicon was digested with *Mlu*I, isolated, then self-ligated to generate pTB110. The pCP-tRNA was used as a template to amplify the promoter of *CpGAPDH* (CPAR2_808670) along with *C. parapsilosis* tRNA^ALA^ with primers PCp808670_FOR and CptRNAAla_REV [29,30]. The amplicon and pTB110 was digested with *Spe*I/*Sac*I and the isolated backbone and the fragment were ligated to create pTB120.

#### pTB121.

The target region for recombination on pADH147 was replaced by amplifying the downstream sequence of the *HIS1* locus (CPAR2_100200) of *C. parapsilosis* CLIB214 (*CpHIS1*) with primers 111-II_FOR and 111-II_REV from *C. parapsilosis* CLIB214 genomic DNA (Fragment 111-II) [24,34]. Primer pairs 111-I_FOR – 111-I_REV and 111-III_FOR – 111-III_REV were used to gain Fragment 111-I and fragment 111-II respectively from the pADH147. The fragments 111-I/II/III were isolated from an agarose gel, and fused by PCR using the primers 111-I_FOR and 111-III_REV. The product was digested with *Pvu*I and self-ligated to obtain pTB111. The gRNA scaffold along with the HDV sequence was amplified with primers gRNA_scaffold_FOR and HDV_REV from pCP-tRNA. The amplicon was digested with *Bss*HII/*Cla*I then ligated with *Mlu*I/*Cla*I digested pTB111 backbone (*Bss*HII and *Mlu*I provide compatible ends) to generate pTB121_no_ter. The terminator of CpGAPDH (CPAR2_808670) was amplified with primers TCp808670_FOR and TCp808670_REV using pCP-tRNA as a template [29,30]. The amplicon and the pTB121_no_ter were digested with *Cla*I and *Hind*III and ligated together to gain pTB121.

#### Templates for donor DNAs.

CpNEUT5L upstream and downstream sequences were amplified from *C. parapsilosis* CLIB214 genomic DNA with primer pairs CpN5L_Up_FOR - CpN5L_Up_REV and CpN5L_Do_FOR - CpN5L_Do_REV respectively, isolated and fused using the primers CpN5L_Up_FOR and CpN5L_Do_REV [34]. The amplicon was isolated and ligated into *EcoR*V/*Stu*I digested pNRVL plasmid backbone to generate pNRVL-N5L [32]. The Promoter – GFP – Terminator sequence was released from pNRVL-S-GFP plasmid with *Eco*RV and *Xho*I and ligated into the pNRVL-N5L at the *EcoR*V/*Sal*I (located between the CpNEUT5L up- and downstream sequences) sites to generate pNRVL-N5L-GFPatt [32]. The *Cla*I/*Nhe*I digested pNRVL-N5L-GFPatt backbone was used in the upcoming ligation reactions. The fluorescent protein coding genes were amplified from pMG1416 (YFP), pMG1683 (CFP), pMG2120 (GFP), pMG2343 (mCherry), pMG2261 (RFP) and the codon optimised derivatives of ffDronpa (ATGme), mScarlet (IDT) and mTurquoise2 (GFPbased) cloned into the pCIp10 with the respective primer pairs listed in S2 Table [35–37]. The amplicons were digested with *Cla*I/*Nhe*I and ligated with the pNRVL-N5L-GFPatt backbone to gain its derivatives carrying the dedicated fluorescent protein coding genes.

#### Generation of donor DNAs.

The pNRVL-N5L carrying the fluorescent protein coding genes was used as a template to amplify the donor DNAs with primer pairs FP_CpN5L_dDNA_F - FP_CpN5L_dDNA_R. Amplicons were purified and concentrated by using PEG-based DNA isolation method [38].

#### Generation of CRISPR/Cas9 components for transformation.

The pTB120 was used as a template to gain the universal “A” fragment with the primers AHO1096 and pTB120univR. The specific “B” fragment was amplified with the primer gRNA_CpN5L or gRNA_Empty and AHO1097 using the pTB121 as a template. The Fragment “A” and “B” were stitched by using PCR with primers AHO1232 and AHO1237 [24]. The Fragment “A”, “B” and “C” were purified with PEG-method right after amplification [38]. The “Cas9” fragment was released by digesting the pTB101 with *Pme*I. Concentration of the fragments was determined according to agarose gel electrophoresis.

#### Transformation.

Plasmids were propagated in the 2T1 strain of *Escherichia coli*. Bacterial transformation was achieved using standard heat-shock method. Cells were plated on LB plates supplemented with the corresponding antibiotics, and incubated overnight at 37 °C. *C. parapsilosis* strains were transformed according to our optimised chemical transformation protocol [39]. 500 fmol “Cas9” fragment, 2500 fmol “C” fragment, and 2500 fmol donor DNA was applied in each transformation. Cells were shaken (160 rpm) for 5 hours in 500 µl YPD at 30 °C after heat-shock, then plated onto YPD-PS plates containing (NTC) in a concentration of 100 µg/ml, and incubated for two days at 30 °C.

#### Excision of the transformation cassette.

PCR validated colonies were inoculated in 300 µl YNB-maltose media, and shaken for approximately one day at 30 °C (160 rpm). The cell concentration was determined by using a Burker-chamber, and ~ 300 cells were plated on YPD plates containing 2 µg/ml NTC. After 2 days of incubation at 30 °C the small colonies were selected. Excision was verified by suspending a small inoculum of the selected strains in 100 µl sterile distilled water, and pinning 5 µl suspension on the top of YPD with or without 100 µg/ml NTC. Plates were incubated at 30 °C, and observed two days later.

#### Validation of the transformants.

Colonies were picked and rapid DNA isolation method was applied according to Holland *et al.* [9]. Integration was tested by three sets of PCR validating the upstream and the downstream site of the integration with primer pairs CpN5LUpChkF–CpOEDoChckF and TDH3DoChkF - CpN5LDoChkR respectively, as well as the overall locus with CpN5LUpChkF and CpN5LDoChkR. Colony PCR verified mutants were further examined with southern-blot according to Gacser *et al.* [8]. Digoxigenin labeled probes were amplified with primer pairs CpN5L_Up_F - CpN5L_Up_R (dDNA integration) or pADH99_fragment2_FOR-pADH99_fragment2_REV primer pairs (transformation cassette integration) from CLIB214 genomic DNA as a template by using alkali-labile Digoxigenin-11-dUTP (Sigma-Aldrich) according to the manufacturer’s instructions. Genomic DNA was digested with *Xho*I (dDNA integration) or *Eco*RI (transformation cassette integration) and the fragments were assayed on 0.8% (m/V) agarose gel along with Roche DIG-labeled DNA Molecular Weight Marker VII.

#### Microscopic imaging: phagocytosis assay.

On the day before the experiment the supernatant of adhered J774.2 cells (~80% confluency) was removed, and the cells were washed with 5 ml DMEM/FBS/PS (37 °C) medium. Two ml fresh medium was pipetted up and down to carefully detach the cells and their concentration was determined by using a hemocytometer. The collected phagocytes were diluted to 10^6^/ml in DMEM/FBS/PS (37 °C), and 100 µl suspension was pipetted into the wells of an ibiTreat 8 well Ibidi µ -Slide, and it was kept in an incubator (37 °C, 5% (V/V) CO_2_ and 100% (V/V) relative humidity) for 16 hours. *C. parapsilosis* strains were prepared as described above, and their concentration was set to 1.25x10^6^-1.25x10^6^-1.25x10^6^/ml for each strain in DMEM/FBS/PS in a common microcentrifuge tube. The supernatant of J774.2 cells was removed, and one hundred µl of the yeast suspension was added. The cells were incubated for three hours at 37 °C, 5% (V/V) CO_2_ and 100% (V/V) relative humidity. Supernatants were removed, the cells were carefully washed with prewarmed (37 °C) 1x PBS then immediately inspected with a Leica Stellaris laser scanning confocal microscope (Leica Microsystems CMS GmbH, Germany) in spiral scan mode using Leica LAS X Navigator software and HC PLAPO CS2 20 × (N.A. 0.75) dry objective. Laser excitation and emission ranges were set either using fluorochrome database of the microscope software or manually, based on the related excitation and emission ranges of the analysed fluorescent proteins. Line sequential mode of capturing was used to prevent crosstalk between fluorescent emissions. Transmitted light images were also collected using transmitted light detector. For each colour-strain combination, two biological replicates were applied per experiment and one picture (approximately 2.5-3 mm^2^) was taken per biological replicate. Two areas each containing 500 macrophages and the uptaken fungi were counted strain by strain per picture and the ratio of the uptaken strains relative to the total number of uptaken fungi was calculated for each area. The experiment was performed twice in three colour-strain combinations. The graphs show the percentages with the standard deviations. Significance levels were calculated by using the Mann-Whitney test (ns: not significant, * : p < 0.05; **: p < 0.01; ***: p < 0.001; ****: p < 0.0001).

#### Microscopic imaging: adhesion assay.

Yeast cells were cultivated and prepared as described above and the cell concentration was set to 2.5x10^6^/ml in 1x PBS in which the ratio of the three fungal strains was 1:1:1. One hundred µl was gently pipetted onto the top of the sterile silicone sheet and incubated for 2.5 hours at 30 °C in a humidified chamber protected from light. The drop was carefully removed and the silicone sheet was washed by gently dipping it three times in sterile 1x PBS. The samples were observed immediately with a Leica Stellaris 5 laser scanning confocal microscope as above. Two biological replicates were applied per experiment and two pictures were taken per biological replicate. The experiment was performed four times per colour-strain combination and all three colour-strain combination were implemented. The yeast cells were counted strain by strain on each picture in an area of approximately 6 mm^2^. The ratio of the given strain in one area was calculated relative to the total number of fungi. The graphs show the percentages with the standard deviation. Significance levels were calculated by using the Mann-Whitney test (ns: not significant, * : p < 0.05; **: p < 0.01; ***: p < 0.001; ****: p < 0.0001).

#### Flow-cytometry.

The yeast cells were grown, harvested and washed as described above. After the second wash with 1x PBS approximately 5x10^6^ cells were suspended in half ml of 4% (V/V) paraformaldehyde and incubated at room temperature for five minutes. Cells were collected, washed twice (2,800 g, 5 min) and suspended in 1 ml 1x PBS. The samples were assayed by using a Beckman Coulter CytoFLEX S Flow Cytometer. As a control 1x PBS and wild-type isolates of the three strains were used to set up the gates in each channel.

#### Growth-curve assay.

The strains were prepared as mentioned above but the wash and suspension steps were carried out in sterile distilled water instead of 1x PBS. The cell concentrations were set to either 5x10^6^/ml in distilled water or 2.5x10^6^/ml in DMEM/FBS/PS. The experiments were performed in a 96 well plate. One hundred µl of the cell suspension in distilled water was pipetted to one hundred µl 2x YPD/PS, while 200 µl of cell suspension in DMEM/FBS/PS was transferred to the wells. As a control, media free of cells were used. Four statistical parallels per strain were applied for both media. The optical density at 600nm was measured every half an hour for 48 hours in each well in a BioTek Synergy HTX microplate reader. The incubation temperature was either 30 °C (YPD/PS) or 37 °C (YPD/PS and DMEM/FBS/PS). Data was evaluated by subtracting the optical density values of the related cell-free media from the ones with the cells. The average of the four statistical parallels with the standard deviation are presented. The experiment was performed three times.

#### *In silico* analysis of adhesin sequences.

The adhesin encoding ORFs of CDC317 were downloaded from candidagenome.org [40]. The sequence of *CpALS4780* from the database was supervised according to the Sanger sequencing results of Oh and coworkers [41]. The ones of GA1 and CLIB214 were obtained from on-line databases according to and Pryszcz *et al.* and Cillingová *et al.* [42,43]. Nucleotide sequences were translated with the translate tool of Expasy using “Alternative yeast nuclear” genetic code [44]. Alignments were generated with MEGA v11.0.13 using MUSCLE algorithm with basic settings [45].

#### Data evaluation.

The microscopic images of phagocytosis were evaluated by using Leica Application Suite X v3.7.6.25997. Fungi from the adhesion assay were counted by using ImageJ 1.54f. Flow-cytometer data was evaluated with CytExpert v2.4.0.28 software. Pictures were edited with GNU Image Manipulation Program v2.8.18. Nucleotide sequences were visualised with “A plasmid Editor” v2.0.51. GraphPad Prism v6.01 was used for statistical analysis and presenting the related data.

## Results

### Overview and adaptation of the plasmid based *C. albicans* CRISPR/Cas9 approach to *C. parapsilosis*

To enable nucleotide precise, markerless, cloning-free application of the CRISPR/Cas9 procedure in *C. parapsilosis*, the technique developed by Nguyen and coworkers for *C. albicans* was modified. This method employs the Cas9 fragment that can be released from a plasmid by digestion, and the Fragment “C” which is a fusion PCR product of the universal Fragment “A” and the specific Fragment “B”. The recombination between the Cas9 and the Fragment “C” occurs at the site of the NAT selection marker, ensuring that only cells that have undergone this process acquire resistance against the antibiotics NTC. After integrating into the genome, the components of the CRISPR/Cas9 system can be expressed and in the presence of the dDNA possessing homologous sequences to the target region, the repair of the Cas9 induced double strand break can be biased towards the homologous directed repair resulting in the intended genetic modification. The excision of the cassette can be achieved in two ways depending on the construct and the site of the integration. In the case of the *Candida maltosa* LEUpOUT or the *C. albicans* LEUpOUT system the loss of the cassette occurs randomly by recombination via the repetitive sequences carried at the two ends of the cassette. In contrast, the HIS-FLP system utilises the maltose inducible flipper system for marker recycling [24,46]. Out of the three possible approaches, we decided to apply the HIS-FLP method in *C. parapsilosis* for multiple reasons. First, we preferred the inducible nature of marker excision over a randomly occurring event. Second, however the double auxotrophy complementation based gene deletion system has been developed for *C. parapsilosis* (that utilises the *C. maltosa LEU2* marker), far less mutants have been generated compared to *C. albicans* [9,47]. Third, only this setup enables any clinical isolate to be genetically altered.

**Fig 1:**
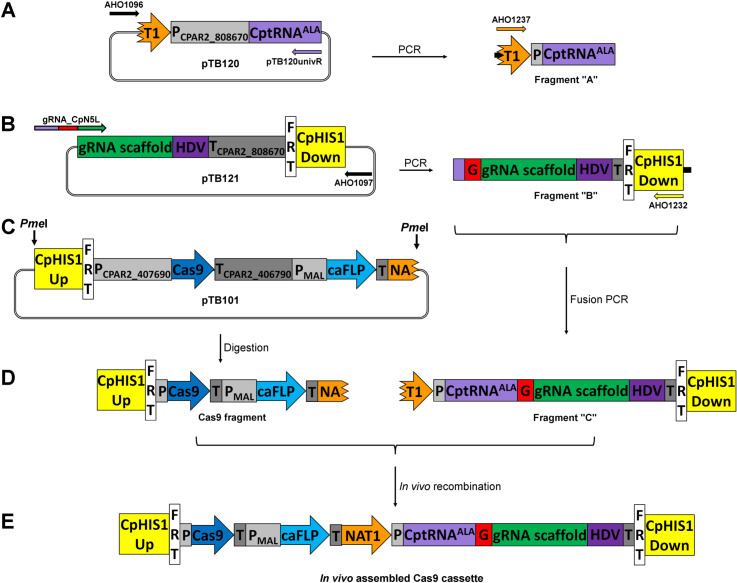
Plasmids and the workflow of the PCR-based CRISPR/Cas9 genome editing approach for *Candida parapsilosis.* The pTB120 serves as a template for generation of the universal Fragment “A” (Panel A). Panel B indicates the amplification of the specific Fragment “B” using the pTB121 as a template with the forward primer carrying the 20 nucleotide long guide sequence (G - red) and a short fusion sequence specific to CptRNA^ALA^ (light purple). Panel C represents the release of the Cas9 fragment by *Pme*I digestion. Panel D describes the recombination between the Cas9 fragment and the fusion PCR product of Fragment “A” and “B” named Fragment “C”. The *in vivo* assembled functional transforming cassette is presented on panel E. The expression of the sgRNA and Cas9 is assured by the native regulatory sequences of CPAR2_808670 and CPAR2_406790 promoter and terminator, respectively. The sgRNA has been equipped with protective sequences of tRNA^ALA^ (light purple) and hepatitis delta virus ribozyme (HDV) (dark purple). CpHIS1 Up and Down are the homologous arms for genomic integration, “T1” (orange): downstream part of the NAT1 selection marker, FRT: flippase recognition target: P_MAL_: maltose inducible promoter, caFLP: codon optimised gene of flippase, “NA” (orange): upstream part of the NAT1 selection marker.

To make the *C. albicans* HIS-FLP system functional in *C. parapsilosis*, the *C. albicans HIS1* target sequences were changed to the ones of *C. parapsilosis HIS1* (CPAR2_100200). Ng and Dean established that the Cas9 editing efficiency could be remarkably enhanced in *C. albicans*, if the level of the intact sgRNA was increased in the cell. This can be achieved by boosting the expression of the sgRNA and providing it with protective sequences at its ends [31]. According to this, we stitched the alanine tRNA (CptRNA^ALA^) to the 5’ end and the hepatitis delta virus ribozyme (HDV) to the 3’ end of the sgRNA. Moreover, this construct was placed under the regulation of the RNA polymerase II related *CpGAPDH3* (CPAR2_808670) promoter and terminator elements, that have been proven efficient in *C. parapsilosis* [29,30]. Additionally, the promoter and terminator sequences of the Cas9 ORF were also adjusted to the ones of *C. parapsilosis* CPAR2_407690 (*CpTEF1*) (Fig 1A, B and C) [29,30]. The components for transformation can be obtained by PCR and digestion in the same way as it was introduced by Nguyen *et al*. The 20 nucleotide long target sequence is stitched to the gRNA scaffold by utilising a primer with the desired target sequence and an additional fusion sequence at its 5’ end. This latter enables the Fragment “B” to be joined with the universal Fragment “A” resulting in the Fragment “C” with the sequence of the functional sgRNA flanked by protective elements: CptRNA^ALA^ and HDV [24]. The Cas9 fragment can be obtained by digesting pTB101 with *Pme*I, and used without purification along with Fragment “C” to transform the fungus, where after recombination and integration into the genome the components can be expressed (Fig 1).

### Generation of fluorescently tagged mutants with the PCR-based CRISPR/Cas9 system

**Fig 2:**
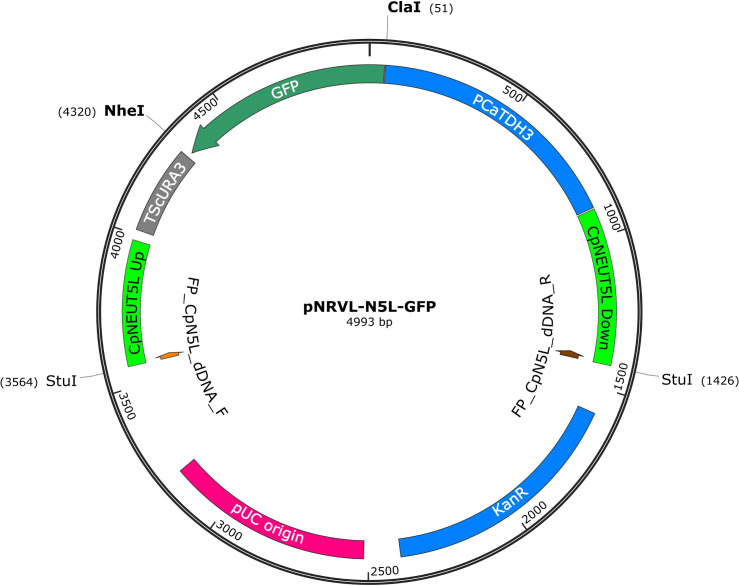
The map of the pNRVL-N5L-GFP plasmid used for dDNA generation. The GFP ORF under the regulation of *C. albicans* TDH3 promoter and *S. cerevisiae* URA3 terminator was equipped with the up and downstream homologous arms of the CpNEUT5L target region. The ORF can be replaced to other genes via *Cla*I/*Nhe*I sites. The dDNA can either be amplified with the highlighted primers (orange and brown arrows), or it can be released by *Stu*I digestion.

As a proof of principle, we aimed to generate a set of fluorescently labeled strains of the CLIB214 clinical isolate [34]. We targeted the CpNEUT5L, a recently described intergenic region that is suitable to accept expression constructs without affecting the stress tolerance of the fungus or its properties upon interaction with J774.2 murine macrophages [32]. To facilitate the dDNA propagation, we introduced the pNRVL-N5L carrying the up- and downstream sites of the CpNEUT5L target locus with the given FP coding genes and regulator sequences in between (Fig 2).

We preferred using longer homologous arms (amplified along with the expression construct) and short primers specific to these, rather than employing large oligos binding to the promoter and terminator with additional 40 (or more) nt long sequences at their 5’ ends with the homologous sequence. This approach not only enables longer homologous arms to use, but also decreases the chance of amplifying unspecific products upon dDNA generation.

**Fig 3:**
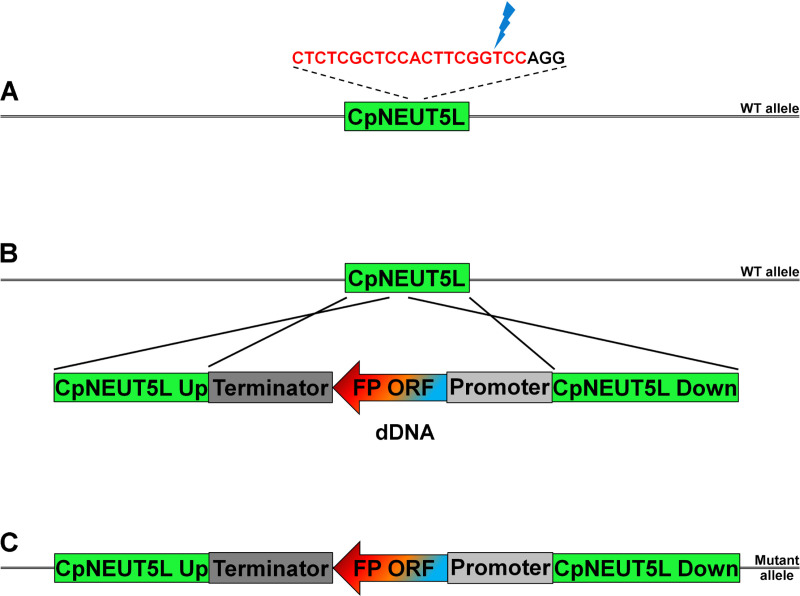
Integration of the dDNA into the genome after Cas9 induced double strand break. Panel A shows the wild-type allele, with the sgRNA target sequence (highlighted in red) followed by the PAM sequence (“AGG”). Blue thunder icon indicates the site of the Cas9 induced double strand break. Panel B presents the dDNA with the given fluorescent protein coding ORF along with the regulatory sequences and its integration by homologous recombination into the target locus. Panel C describes the altered CpNEUT5L locus carrying the expression construct.

First, we tested a total of eight different fluorescent proteins in CLIB214: cyan fluorescent protein (CFP), green fluorescent protein (GFP), mCherry, red fluorescent protein (RFP), yellow fluorescent protein (YFP) and the codon optimised versions of fast folding Dronpa (ffDronpa), mScarlet and mTurquoise2 [35–37]. The dDNAs were amplified from the plasmid templates in a way that the final amplicons consisted of the ORF of the given FP under the regulation of the *C. albicans* TDH3 promoter and *Saccharomyces cerevisiae* URA3 terminator, flanked by the up- and downstream homologous sequences (363 and 414 bp respectively) of the target locus (Fig 3).

Besides transformation mixtures employing Cas9 fragment, Fragment “C” and the different dDNAs, the applied fragments were also used individually as controls and in addition a Fragment “C” called “empty” along with the Cas9 fragment was also involved that contained no sgRNA target sequence between the CptRNA^ALA^ and sgRNA scaffold (Table 1).

**Table 1:** Layout of the transformation reactions with respect to the applied fragments.

		1	2	3	4	5	6	7	8
Fragment “C”	N5L	+	+	–	–	–	+	–	–
	Empty	–	–	+	+	–	–	–	–
Cas9 fragment		+	+	+	+	–	–	+	–
dDNA (mScarlet)		+	–	+	–	+	–	–	–
No. of colonies		24	10	20	13	0	0	0	0
No. of white colonies		10	10	20	13	0	0	0	0

**Fig 4:**
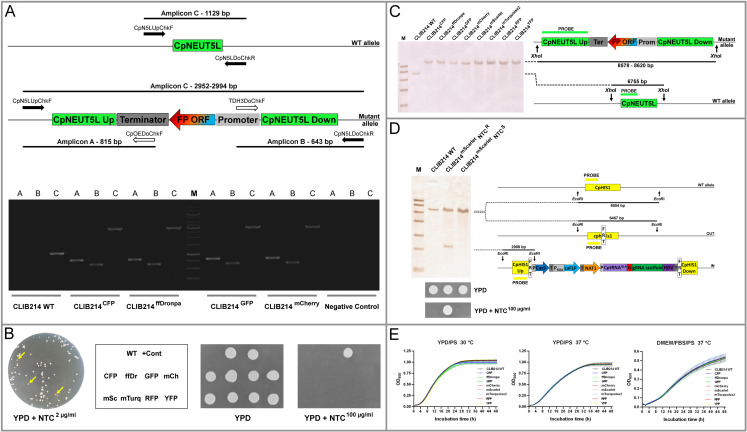
Validation of the FP expression transformants. Panel A presents the scheme of validation and an example (involving CLIB214 WT derived transformants) by using primers specific to the genome solely (solid arrows) or to the integrated construct (empty arrows). Amplicon A and B are present only when the dDNA has been integrated. In contrast, Amplicon C can be gained from the genome of the FP carrying transformants and from the one of the wild-type as well, but the length of the amplicons is distinct by the size of the integrated construct. DNA marker: 10,000; 8,000; **6,000**; 5,000; 4,000; 3,500; **3,000**; 2,500; 2,000; 1,500; **1,000**; 750; 500; 250 bp. Panel B shows colonies in different size after cultivating the PCR confirmed clones in YNB+maltose medium overnight to induce the excision of the transformation cassette. Cells were spread onto YPD plates supplemented with 2 µg/ml NTC and incubated for one days at 30 °C. Smaller colonies (yellow arrows) were taken and pinned onto YPD and YPD supplemented with 100 µg/ml NTC plates to test the FLP catalysed loss of the selection marker (WT: CLIB214 and its FP expressing derivatives, ffDr: ffDronpa, mCh: mCherry, mSc: mScarlet, mTurq: mTurquoise2, + Cont: NTC resistant CLIB214 ^pN-S-GFP^). Panel C describes the validation of NTC sensitive clones with southern-blot using CLIB214 wild-type and all its FP expressing transformants. The size of the amplicons and fragments varies due to the different length of the FP coding ORFs. Panel D highlights the confirmation of the loss of the transforming cassette. The example shows the case of CLIB214 ^mScarlet^. The scheme (on the right) describes the strategy of the southern-blot. NTC test at the bottom involves the same strains in the same order as they are on the filter. NTC^R^: NTC resistant, NTC^S^: NTC sensitive. Panel E presents the growth curve analysis of the NTC resistant FP expressing CLIB214 mutants validated by molecular techniques. The viability of the mutants was tested in YPD/PS (at 30 and 37 °C) and DMEM/FBS/PS (at 37 °C) complete media.

Colonies emerged only when the Cas9 fragment had been utilised along with any of the Fragment “C”s. More colonies appeared when the dDNA was also present in the transformation mixture. We observed NTC resistant transformants with altered colour that was particularly noticeable upon using mScarlet as a dDNA where the colour of the colonies ranged from white through pink to cyclamen. The NTC resistant colonies were first tested with PCR (Fig 4A).

The confirmed ones were cultivated in the presence of maltose to induce the excision of the transformation cassette and then plated on YPD/PS plates containing 2 µg/ml NTC. Two populations differing in size appeared after two days of incubation suggesting that the induced loss of the transformation cassette occurred in many but not all the cells. Small, sensitive colonies were taken to confirm the absence of the selection marker by pinning them onto YPD/PS/NTC^100^ and YPD/PS as a control (Fig 4B). NTC sensitive strains were also analysed with southern-blot that confirmed homozygous integration in all the eight cases (Fig 4C). Additionally, we investigated the *CpHIS1* genomic locus as well with southern-blot that indicated a single copy integration into this region. Moreover, our assay verified that the lack of growth in the presence of 100 µg/ml NTC indeed correlated with the loss of the transformation cassette (Fig 4D).

### Fluorescent tagging does not alter the growth in rich media

To investigate whether the modification affects the proliferation of the verified mutants, we recorded the growth curves for 48 hours by cultivating the cells in YPD/PS at 30 and 37 °C and in DMEM/FBS/PS media (37 °C). We identified mutants with no evidence for any change in the growth of the FP expressing strains compared to that of the parental strain indicating that the expression construct does not alter the viability of the mutants under these circumstances even if it is present in two copies in the genome (Fig 4E).

### Fluorescent imaging of the labeled strains identified mScarlet, mTurquoise2 and YFP suitable for simultaneous use

**Fig 5:**
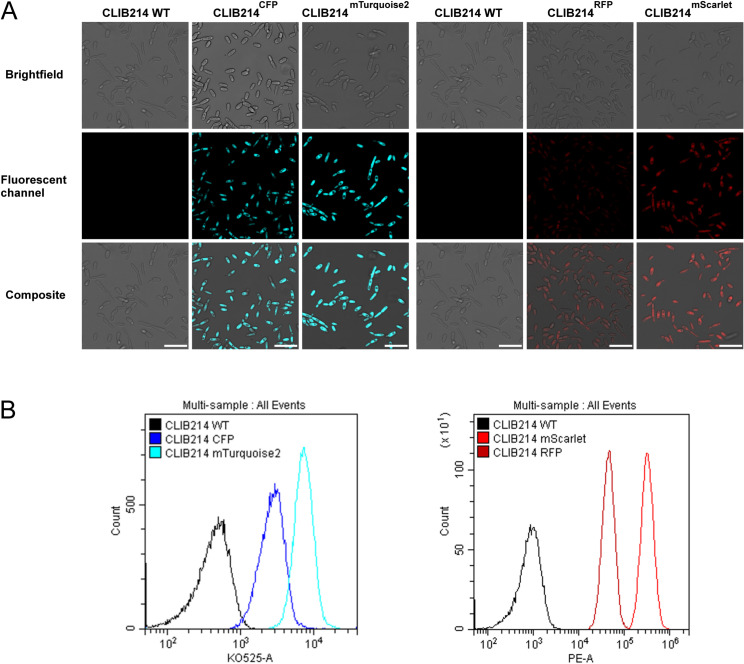
Fluorescence imaging of CFP, mTurquoise2, RFP and mScarlet expressing CLIB214 mutants. Panel A presents microscopic images, Panel B shows the flow cytometer analysis of the related strains compared to the parental strain (x axis indicates the intensity of the fluorescent channel). Scale bar is 20 µm.

Mutants were inspected with a flow cytometer (S1 Fig) and a fluorescence microscope (S2 Fig). We established that more than 98% of the populations expressed the FPs in question except for CFP that was less than 82%. Additionally, we found that the signal of CFP and RFP were very weak compared to the ones of other blue and red light emitting FPs (mTurquoise2 or mScarlet for instance), which phenomena was also confirmed with microscopic observations (Fig 5). Consequently, we excluded these mutants (and FPs) from our further investigations.

**Fig 6:**
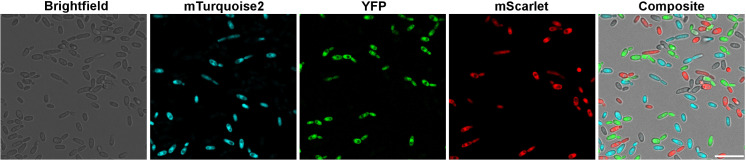
Microscopic imaging of the mix of mTurquioise2, YFP, mScarlet expressing—and the parental CLIB214 strains. By this FP combination the strains were easy to distinguish from each other without interference with other fluorescence channels. Scale bar is 20 µm.

We also aimed to identify a combination of FPs that can be used in the same experiment and distinguished from each other with ease. Although the signal intensity of the GFP was amongst the highest, unfortunately with our setup it was also present in the blue channel therefore this FP needed to be omitted as well (S1C Fig). Using the combination of mTurquoise2, YFP and mScarlet resulted in minimal cross talk between blue, green and red channels (Fig 6).

### Generation of FP expressing mutants in CDC317 and GA1

In order to investigate the intraspecies heterogeneity, two often cited clinical isolates of *C. parapsilosis* were sentenced to fluorescent labelling with mScarlet, mTurquoise2 and YFP. The transformants of CDC317 and GA1 isolates were validated with the same molecular methods and investigated for growth and fluorescent properties as were the CLIB214 FP expressing strains (S3 and S4 Figs). The set of mScarlet, mTurquoise2 and YFP expressing derivatives of the three isolates were applied in competitive assays and their properties with regard to macrophage interaction and adhesion have been investigated.

### GA1 is more robustly uptaken by J774.2 macrophages than CDC317 or CLIB214 isolates

**Fig 7:**
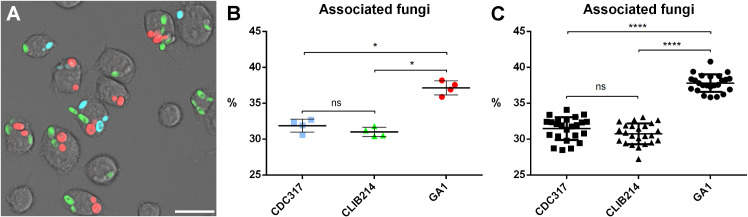
Investigation of the uptake of different *C. parapsilosis* clinical isolates by J774.2 macrophages. Panel A shows a microscopic image of the co-incubation assay three hours after the infection (blue: CDC317 ^mTurquoise2^, green: CLIB214 ^YFP^, red: GA1 ^mScarlet^). The macrophage associated yeasts were counted, and their ratio relative to the total number of macrophage-associated yeasts was determined strain by strain (Panel B). The experiment was performed twice with three colour-strain combination, with two biological parallels and four statistical parallels per experiment. For statistical analysis Mann-Whitney test was used (ns: not significant, * : p < 0.05, ****: p < 0.0001). Scale bar is 20 µm.

To find out if there is any preference in phagocytosis by J774.2 murine macrophages towards any of the three isolates of *C. parapsilosis*, we incubated the phagocytes with the labeled strains in a ratio of 1:3:3:3 (J774.2:CDC317:CLIB214:GA1). The microscopic images taken after three hours of incubation were evaluated. The yeast cells uptaken by the macrophages were counted strain by strain. Being aware of the total number of phagocytosed fungi, we determined the percentages of uptaken fungi strain by strain relative to the total number of phagocytosed fungi. This revealed that significantly higher percentage of the cells of the GA1 isolate (37.8 ± 1.2%) was phagocytosed by the macrophages than either CDC317 (31.5 ± 1.6%) or CLIB214 (30.7 ± 1.5%) were, between of which there was no significant difference (Fig 7).

We performed the experiment by changing the colour – strain combinations, and all these setups led to similar findings, showing that these results are indeed related to the nature of the strain and not to the FP applied (S5 Fig).

### CLIB214 is less adherent to silicone material than CDC317 or GA1 strains

**Fig 8:**
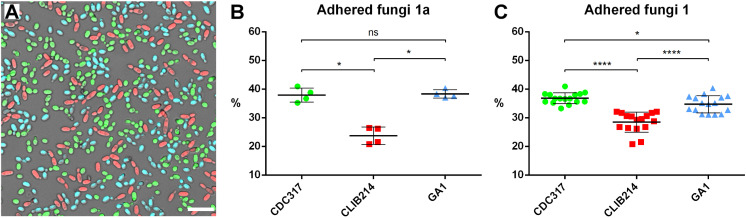
Characterisation of the adhesion capacity of the *C. parapsilosis* isolates to silicone. The strains were mixed in a 1:1:1 ratio and let to adhere to the silicone surface for two and a half hours and observed with a fluorescence microscope (green: CDC317 ^mTurquoise2^, red: CLIB214 ^YFP^, blue: GA1 ^mScarlet^) (Panel A). The cells were counted and their ratio was calculated strain by strain (Panel B). The experiment was performed four times per colour-strain combination with two biological and a total of four statistical parallels in each experiment. The experiment was assessed for each colour-strain combination (Panel C). Statistics was calculated according to Mann-Whitney test (ns: not significant, * : p < 0.05, ****: p < 0.0001). Scale bar is 20 µm.

As *C. parapsilosis* is often associated with medical devices such as intravenous catheters known as a predisposing factor for systemic candidiasis and eventually outbreaks of infections, we tested the adherence of the FP labeled strains to silicone by mixing them in a 1:1:1 ratio and letting them adhere to this material for two and a half hours [48]. Microscopic images were taken and assessed to establish the ratio of the given strain compared to the total number of adhered fungi in a way similar to the phagocytosis assay (Fig 8).

We identified CDC317 the most adherent and CLIB214 the least adherent strain. The performance of the GA1 however was not obvious, it ranged between the two other strains from experiment to experiment, but it was not a colour dependent phenomenon. For a more precise analysis we doubled the number of biological replicates and evaluated them for each colour-strain combination separately. The three independent colour-strain combinations were consistent all strengthening the dominance of CDC317 (36.8 ± 0.5%, 40.4 ± 0.6, 38.1 ± 0.5), followed by GA1 (34.7 ± 0.7%, 32.7 ± 0.8%, 35.0 ± 0.6%) and classifying CLIB214 (28.5 ± 0.9%, 27.0 ± 1.1%, 26.9 ± 0.9%) as the least adherent of the three strains (S6 Fig). The most extensively studied role players in *Candida* adhesion are the Agglutinin-like sequences (*ALS*s). Given that all these isolates have only one *ALS* gene (*CpALS660*) in common and CDC317 and CLIB214 have four (*CpALS4770*, *CpALS4780*, *CpALS4790*, *CpALS4800*) while GA1 has only one additional (showing high similarity with *CpALS4800*), the dominance of GA1 over CLIB214 was surprising [4,41–43]. In order to come up with a hypothesis explaining our results we performed a protein sequence analysis according to the available genome sequence of the three strains regarding the *ALS* genes (S4 Table).

### Sequence analysis of the selected Als proteins

The Als sequences of the *C. parapsilosis sensu lato* group were thoroughly analysed and characterised *in silico* in recent studies [41,49]. Here we aim to highlight only the differences between the same genes/proteins from the different isolates.

Both CpAls4770 and CpAls4780 carry SSSEPP tandem repeats. This motif however slightly truncated or altered in both isolates (to SSEPP or more commonly SASEPP in CpAls4770 and to SSSESE or SSSEPE in CpAls4780 for instance). The only difference we identified between the two strains in the protein sequence of CpAls4770 was a lack of a single SASEPP motif in CDC317 compared to CLIB214. Interestingly, this protein encoded more SASEPP (or SASEPS) than SSSEPP motif in general. In contrast, CpAls4780 carries predominantly SSSEPP motifs occasionally segmented by SSSEPE, SSSESE, SSSETE, SSSGPP and SSTEPP sequences out of which a total of 39 copies were identified in CDC317. Three complete SSSEPP motifs are missing in the CpAls4780 of CLIB214. Unlike CpAls4770, CpAls4780 encodes two other types of repetitive sequences. The GSGN (or sometimes GSGE) motifs are located in the C-terminal region of the protein and are present in 46 copies in the two strains according to the NGS data [40,43]. One copy of these however, might be the consequence of an imperfect assembly as Sanger sequencing failed to verify an ATCAGGAGAAGG nucleotide sequence (one GSGE motif) in the position 3228-3239 in CDC317 (according to NGS data) [41]. This might be true also for CLIB214. The other characteristic repetitive sequence was a PTTTDNGNGGENT motif, that maps closer to the central part of the protein and exists in 19 exact copies in CLIB214, which is 8 more than in CDC317. Contrary to CpAls4770, CpAls4780 proteins showed several other strain dependent alterations represented by single or double amino acid differences.

The CpAls4790 and CpAls4800 are similar in terms of their central domain consisting of tandemly repeated sequences and a serine and threonine rich C-terminal region [41]. There are two dominant (slightly similar) repetitive motifs in CpAls4790, both consisting of 36 amino acids that compose the central ~1500 amino acid long sequence of the protein. CLIB214 encodes three more of these than CDC317 does. Besides this, very few amino acid differences exist between the two strains in the amino acid sequence of the CpAls4790 proteins.

The CpAls4800 is shorter than CpAls4790 that is mostly due to the lesser copies of a 36 amino acid long repetitive sequence, out of which CDC317 encodes one more than CLIB214 does. Very few amino acid alterations that are present between the protein sequence of the two isolates affect only the above mentioned motif.

We previously reported that in GA1 an approximately 23.5 kbp region has been lost compared to the reference CDC317 genome very likely due to a single recombination event that affected the region of the four neighbouring *ALS* genes (*CpALS4770*, *CpALS4780*, *CpALS4790*, *CpALS4800*) [42]. The complete deletion of *CpAls4780* and *CpAls4790* has presumably been initiated by sequence similarity between *CpALS4770* and *CpALS4800* [42]. The breakpoint maps between the 391st and 392nd nt of the fused gene of GA1 resulting in the formation of a 3072 nt long ORF. The sequence upstream from the breakpoint is completely identical with the upstream sequence of *CpALS4770* of CDC317 without any difference in the nucleotide sequence. The remaining 2681 nt shows high similarity to the downstream part of *CpAls4800*. This region carries a total of 14 copies of a 108 nt long sequence in CDC317 (13 in CLIB214) out of which only 4 copies are present in the fused sequence of GA1 (ranging from the 1333rd to the 1764th nt) with only four nt difference at this site relative to CDC317. Besides the number of this repeat, only a single nucleotide difference could have been recognised between the downstream part of *CpALS4800* from CDC317 and the newly formed ORF of GA1. To note, the upstream part of *CpAls4780* also carries a sequence similar to the one of *CpAls4770* and *CpAls4800* raising an opportunity for recombination and eventually emergence of new sequence combinations in other *C. parapsilosis* isolates.

*CpALS660* is the only *ALS* gene that is shared amongst all the three isolates and the encoded protein resembles CpAls4780 in its structure. It is characterised by complete and incomplete motifs of SSSEPP in the middle, followed by tandem repeats of a PTTTDNGNGGENT sequence and abundance of GSGN motifs at the C-terminal end. CLIB214 carries an extra SSSGPP compared to GA1 and CDC317, while at another site GA1 and CLIB214 miss a SSSGPP-SSSEPP-SSSEPP-SSSESE-SSSEPP sequence. Only four PTTTDNGNGGENT complete (and an incomplete PTTTDNGNGGKNT) motifs were found in CLIB214, while the other two isolates lack two copies of this motif. A total of 41 copies of GSGN (or GSGE) sequences were identified in both CDC317 and GA1 that were completely identical. In contrast, there are only 10 copies of this motif in CLIB214. Few differences in the amino acid sequence between the CpAls660 of the different isolates occurred in the SSSEPP repeat region.

### Performance of the PCR based CRISPR/Cas9 system

The obvious alteration in the colour of the transformants upon using mScarlet as the dDNA provided a great opportunity to characterise the performance of this CRISPR/Cas9 method (Table 1). All these types were sentenced to PCR and we found a large heterogeneity amongst the 24 NTC resistant colonies tested. Twenty of the 24 colonies have undergone genetic alteration in the examined CpNEUT5L locus and four remained intact in this region according to PCR (S7A and S8A Figs). All twenty clones yielded amplicon “A” and “B”, but the expected size of amplicon “C” appeared in only nine cases, a larger amplicon “C” was present in four cases and no amplicon “C” could have been generated from seven samples. Southern-blot analysis of the 24 NTC resistant mutants indicated that in line with the PCR four remained unaffected in the CpNEUT5L locus and only five out of the nine PCR confirmed clones were found appropriate (S7B and S8B Figs). All the clones were tested with flow cytometry as well and we observed that the fluorescent intensity of some of the mutants not confirmed with southern-blot was an order of magnitude higher than the that of the validated ones and it was also present not only in PE (red), but also in the FITC (green) and KO-525 (blue) channels (S7D and S8D Figs).

**Fig 9:**
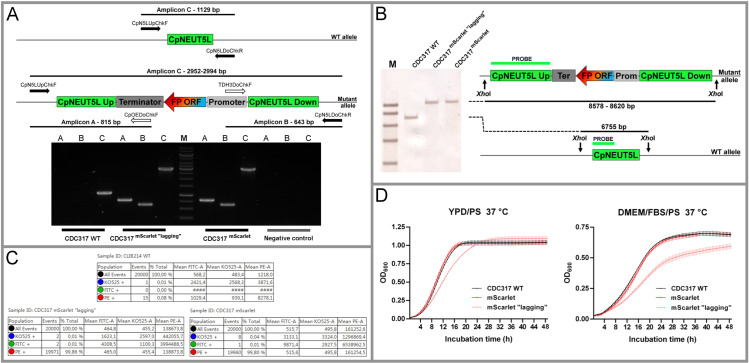
Characterisation of the mScarlet expressing derivatives of CDC317 strain. Panel A and B show the confirmation of two independent CDC317 ^mScarlet^ clones by molecular methods. DNA marker: 10,000; 8,000; **6,000**; 5,000; 4,000; 3,500; **3,000**; 2,500; 2,000; 1,500; **1,000**; 750; 500; 250 bp. Panel C indicates their fluorescent properties according to flow cytometry. Panel D presents the results of the growth curve assays.

We faced one case when the removal of the transforming cassette was unsuccessful. This phenomenon occurred upon generating the CDC317 ^mTurquoise2^ strain. Another issue has been recognised when the growth of the PCR and southern-blot verified mScarlet expressing CDC317 mutants was monitored. These mutants were identical in their fluorescent properties as well but either of them showed a noticeable lag when cultivated in YPD/PS or DMEM/FBS/PS compared to the parental strain (Fig 9).

As several other candidates had been generated, we selected another CDC317 ^mTurquoise2^ and CDC317 ^mScarlet^ strain not having the above mentioned problems.

## Discussion

The primary goal of our work was to enable rapid fluorescent labelling of prototroph *C. parapsilosis* isolates by introducing a PCR-based CRISPR/Cas9 method. This technique was successfully applied in all three clinical isolates we tested that enabled us to characterise their virulence related aspects in competition assays. We customised an already existing method originally designed for *C. albicans* and integrated eight different FP coding genes into our dDNA generating workflow. To facilitate the generation and validation of such mutants in *C. parapsilosis* we provide a complete step by step protocol (S1 Appendix). This system was suitable to generate homozygous knock-in mutants in three prototroph isolates with clinical relevance in just one round of transformation. Modification of the neutral intergenic CpNEUT5L region of *C. parapsilosis* did not alter the growth of the mutants compared to the control isolates in rich media. In our case the editing efficiency was above 80% as twenty out of the 24 investigated clones had alteration in the CpNEUT5L region (when applying mScarlet dDNA on CLIB214 background). This is permitted by the high efficient activity of the endonuclease that consequently enables the selection marker to be placed on the plasmid or the integrating construct rather than the dDNA which is the greatest advantage of the most recent CRISPR/Cas9 systems over the previous genome modification approaches or early CRISPR/Cas9 methods, where an exogenous sequence (FRT, dominant or auxotrophic selectable marker) was always left behind at the altered genomic site raising concerns regarding if the phenotype of the mutant is due to the intended modification or it is the consequence of the exogenous sequence itself [8,9,22,24,29,30]. The nature of the CRISPR/Cas9 method however brings up the issue of the so-called off-target effect which means an unintentional modification in an untargeted region likely due to the presence of a sequence with high similarity to the sgRNA targeted sequence. Additionally, decades of experience suggests recombination hotspots in the genome of *C. parapsilosis* that further increases the chance for ectopic integration of the dDNA. This is what we investigated when the mScarlet dDNA was used where the fluorescent intensity of CLIB214 ^mScarlet^ strains varied greatly and was present in fluorescent channels other than PE in some cases. This correlated well with the results of the southern-blot that revealed ectopic or multicopy integration in robust mScarlet expressing clones instead or besides CpNEUT5L. In total, out of the nine PCR confirmed transformants only five were verified also by southern-blot demonstrating that this is an absolutely indispensable method for thorough mutant validation. Two issues emerged when transforming the CDC317 strain: one in terms of marker recycling the other when the growth of a PCR and southern-blot validated mutant differed significantly from its parental strain. The reason for these particular phenomena has not been investigated further, as several other confirmed clones were available. Due to the limited number of mutants generated so far, we also cannot conclude if this is a strain dependent feature or it can occur also in CLIB214 and/or GA1.

Although CFP and RFP were applied with great success in *C. albicans* these provided weak fluorescent signal in *C. parapsilosis* and codon-optimised version of mTurquoise2 and mScarlet, besides mCherry are recommended instead [35–37,50]. We found that GA1 was significantly more frequently uptaken than CDC317 or CLIB214 by J774.2 macrophages which is in line with the findings of Tóth *et al.* who established a preference of J774.1 macrophages towards GA1 over CLIB214 in a phagocytosis assay, however these two strains were identified according to their shape (GA1 is spherical, CLIB214 is elongated) [51]. The combination of mScarlet, mTurquoise2 and YFP permitted us to perform competition assays with three strains for the first time enabling differentiation even between CDC317 and GA1 that are impossible to distinguish from each other by their appearance. In our adhesion assay CDC317 was the most while CLIB214 was the least adherent to silicone surface with GA1 fitting between the two. Adhesion enables biofilm formation and eventually colonisation of a given surface. Considering the initial steps, the nature of the cell wall and the quality and the quantity of the cell wall proteins can act as relevant factors. Unfortunately, in contrast to *C. albicans*, which is extensively studied in terms of adhesion and related factors like gene expression patterns, protein structure and ligands, much less data is available in *C. parapsilosis* with respect to this phenomenon [52,53]. The biofilm formation capacity of *C. parapsilosis* isolates shows a large diversity that interestingly, can be predicted according to the morphology of the colony as the non-smooth colony morphotype shows a strong correlation with a better biofilm formation capacity [54]. These two features also couple with the abundance of cell wall attached adhesins [55]. In *C. albicans* there are three families of cell wall proteins that are considered as main contributors to adhesion: the Iff/Hyr family, the Hwp family and the Als family [56]. The genome of *C. parapsilosis* comprises many orthologs of these genes whose expression was found to be more dependent on the macro- and micromorphology of the strain rather than on the cultivating conditions [55]. Experimental data regarding *C. parapsilosis* adhesion is limited to the five *ALS* genes that most isolates carry. These studies revealed that all five Als proteins play a role in adhesion *in vivo* in a murine vaginal or urinary tract infection, however *in vitro* only CpAls4770, CpAls4790 and CpAls4800 seemed to be involved in the adherence to human buccal epithelial cells (HBECs) *in vitro*. The disruption of the single genes one by one had no effect on the expression of the intact *ALS*s. In contrast, when both *CpALS770* and *CpALS4780* were disrupted the adhesion to HBECs as well as the biofilm forming capacity of the mutant increased significantly, coinciding with the elevated expression of *CpALS4790* that might yet reflect to the existence of an underlying compensation mechanism [57–59]. The dominance of GA1 over CLIB214 in our assay was an unexpected phenomenon as CLIB214 and CDC317 both encode a total of five *ALS* genes, while only two have been identified in GA1 [41,42]. This recognition led us to analyse the nucleotide and protein sequences of these genes in more detail, revealing significant differences amongst the isolates affecting the number of repetitive sequences (besides minor amino acid alterations) that is in line with the recent findings on *CpALS* genes [41]. Moreover, we found an evidence for a formation of a new *ALS* ORF by intergenic recombination in GA1. Similar phenomena have already been described in *C. albicans* where the recombination between *ALS5* and *ALS1* led to the formation of *ALS51* [52,60].We concluded that the gene earlier identified as *CpALS4800* in this isolate might not be an actual allelic variation of the *CpALS4800* from the reference strain [41]. We suppose that the reason for the high similarity is that nearly 90% of the newly formed gene in GA1 is highly similar to the downstream part of *CpALS4800* from CDC317 while the short 5’ end of the gene with the related promoter comes from *CpALS4770* suggesting that the regulation of the new gene is likely to be similar to the one of *CpALS4770* in CDC317. Due to the feasible ancestry of this gene in GA1 we propose the application of the nomenclature *CpALS477480*, indicating its relationship with *CpALS4770* and *CpALS4800*. We have not performed any functional experiments related to *ALS*s as it was beyond the original scope of this study however, we believe that our *in silico* findings raised several questions to be answered in the future especially focusing on if the sequence or the number of a dedicated repeat plays any role in the adhesion and how.

A recently revealed issue with the CRISPR/Cas9 system in *C. parapsilosis* is the development of aneuploidy and loss of heterozygosity even hundreds of kilobases away from the Cas9 induced DSB [61]. To make solid scientific statements the application of more independent replicates of each strain is recommended in the experiments that, if they behave in a same way, strengthen the role of the investigated gene and the related protein. To follow this idea in our experiments the FP expressing clinical isolates were tested with PCR, southern-blot and for growth as well, and were always applied in a colour-strain rotation pattern which finally resulted in the utilisation of three independent mutants per isolate and three different setups. As all these setups independently from each other led to similar results in each experiment we consider our mutants and statements valid. According to our results we would like to extend the suggestions on proper application of the CRISPR/Cas9 technique. First, if the application of Eukaryotic Pathogen gRNA Design Tool is possible, then it is suggested to do so especially focusing on minimising the off-target effect [62]. It is also advised to remove the Cas9 from the cell as soon as possible. As it was proposed before, it is recommended to apply more independent clones for phenotypisation and generation of proper reintegrant strains (where possible) seems to be mandatory in the CRISPR/Cas9 era as well [61]. If the nature of the modification allows southern-blot validation the ectopic integration of the dDNA can be revealed. Monitoring the growth curve of the transformants with their respective control can also indicate unintended alteration(s). If accessible, NGS sequencing could be the most accurate screen, however it is not affordable for every laboratory.

Along with the plasmid based and the current, integrating approach there are already two different CRISPR/Cas9 methods available for *C. parapsilosis* genome editing so one can decide if the ligation or the PCR based version is more preferred. The proper combination of fluorescent proteins opens up the possibility to follow up to three different strains of *C. parapsilosis* in an assay under the very same conditions. To aid this we provide a detailed protocol for fluorescently labelling *C. parapsilosis* for an in-depth understanding of the pathogenicity of this human pathogen.

## Supporting information

S1 Fig Analysis of the FP expressing CLIB214 strains by flow cytometer. The parental strain was used to set up the proper gate (Panel A). The KO525 channel was applied for CFP and mTurquoise2 (Panel B), ffDronpa, GFP and YFP were detected in FITC channel (Panel C), and PE was used to detect the fluorescence of mCherry, mScarlet and RFP (Panel D). On the histograms the fluorescence intensities (x axis) of the FP expressing strains are compared to the one of parental strain. Statistical analysis of each mutant regarding the fluorescence in each channel is summarised below the given histograms.
(TIF)

S2 Fig Microscopic analysis of ffDronpa, GFP, mCherry and YFP expressing mutants of the CLIB214 isolate. (TIF)

S3 Fig Investigation of FP expressing derivatives of CDC317 isolate by molecular methods and fluorescent imaging. Panel A and Panel B show the validation by using PCR and southern-blot respectively. Panel C presents an example of the gating and the histograms using the parental strain as a reference in the given fluorescent channel. The statistics of the fluorescent signal is summarised below each histogram. Panel D summarises the microscopic images of the investigated strains. Panel E introduces the growth curves of the CDC317 isolate and its FP expressing strains grown in YPD/PS medium at 30 and 37 °C, and DMEM/FBS/PS medium at 37 °C.
(TIF)

S4 Fig Investigation of FP expressing derivatives of GA1 isolate by molecular methods and fluorescent imaging. Panel A and Panel B show the validation by using PCR and southern-blot respectively. Panel C presents an example of the gating and the histograms using the parental strain as a reference in the given fluorescent channel. The statistics of the fluorescent signal is summarised below each histogram. Panel D summarises the microscopic images of the investigated strains. Panel E introduces the growth curves of the GA1 isolate and its FP expressing strains grown in YPD/PS medium at 30 and 37 °C, and DMEM/FBS/PS medium at 37 °C.
(TIF)

S5 Fig Individual evaluation of the co-incubation assay experiments. The J774.2 macrophage associated yeasts were counted and their ratio relative to the total number of macrophage associated yeasts was determined strain by strain. The shape of the signs indicate the strain (square: CDC317, triangle: CLIB214, circle: GA1), their colour refers to the applied FP in the given experiment (green: YFP, red: mScarlet, blue: mTurquoise2). The numbers in the titles illustrate the colour combination (columns), the “a” and “b” letters highlight the two repeats per colour-strain combination. One experiment was performed in biological duplicates, and two statistical parallels were applied per biological sample. Statistical analysis was performed according to Mann-Whitney test (ns: not significant, * : p < 0.05).
(TIF)

S6 Fig Individual evaluation and the summary of the adhesion assay experiments. The yeasts adhered to silicone were counted, and their ratio relative to the total number of yeasts was determined strain by strain. The shape of the signs indicate the strain (square: CDC317, triangle: CLIB214, circle: GA1), their colour refers to the applied FP in the given experiment (green: YFP, red: mScarlet, blue: mTurquoise2). The numbers in the titles illustrate the colour combination (columns), the “a”, “b”, “c” and “d” letters indicate the four biological repeats per colour-strain combination. One experiment was performed in biological duplicates and two statistical parallels were applied per biological replicate. The results were summarised per colour-strain combination. Statistical analysis was performed according to Mann-Whitney test (ns: not significant, * : p < 0.05; ***: p < 0.001; ****: p < 0.0001).
(TIF)

S7 Fig Investigation of the performance of the PCR-based CRISPR/Cas9 technique applying mScarlet dDNA. FP expressing derivatives of CLIB214 (1-12) were characterised by molecular methods and fluorescent imaging. Panel A and Panel B show the validation by using PCR and southern-blot respectively. The investigated transformants were pinned and cultivated on YPD/PS plate, and are presented under the filter. Panel C introduces the statistics of flow cytometry using the parental strain as a reference and the FP expressing CLIB214 ^mTurquoise2^, and CLIB214 ^YFP^ for gating. Black line: WT/unaffected strain according to PCR and southern-blot, green line: PCR and southern-blot confirmed mutant, yellow line: mutant confirmed with PCR, but not with southern-blot, orange line: mutant not confirmed by PCR (Size of Amplicon “C” is not correct) and southern-blot.
(TIF)

S8 Fig Investigation of the performance of the PCR-based CRISPR/Cas9 technique applying mScarlet dDNA. FP expressing derivatives of CLIB214 (13-24) were characterised by molecular methods and fluorescent imaging. Panel A and Panel B show the validation by using PCR and southern-blot respectively. The investigated transformants were pinned and cultivated on YPD/PS plate, and are presented under the filter. Panel C introduces the statistics of flow cytometry using the parental strain as a reference and the FP expressing CLIB214 ^mTurquoise2^, and CLIB214 ^YFP^ for gating. Black line: WT/unaffected strain according to PCR and southern-blot, green line: PCR and southern-blot confirmed mutant, yellow line: mutant confirmed with PCR, but not with southern-blot, orange line: mutant not confirmed by PCR (Size of Amplicon “C” is not correct) and southern-blot, red line: mutant not confirmed by PCR (Amplicon “C” is missing) and southern-blot.
(TIF)

S1 Appendix Protocol for fluorescent labelling of *C. parapsilosis* cells with the PCR-based CRISPR/Cas9 method. (PDF)

S1 Table List of strains used in this study. (XLSX)

S2 Table List of oligonucleotides used in this study. (XLSX)

S3 Table List of plasmids used in this study. (XLSX)

S4 Table CpAls protein sequences. (XLSX)
