# Supplementary figures and images for: PCR-based CRISPR/Cas9 system for fluorescent tagging: A tool for studying Candida parapsilosis virulence
Source: PLoS One. 2025 Feb 24;20(2):e0312948. doi: 10.1371/journal.pone.0312948 (PMC12338950; doi:10.1371/journal.pone.0312948)

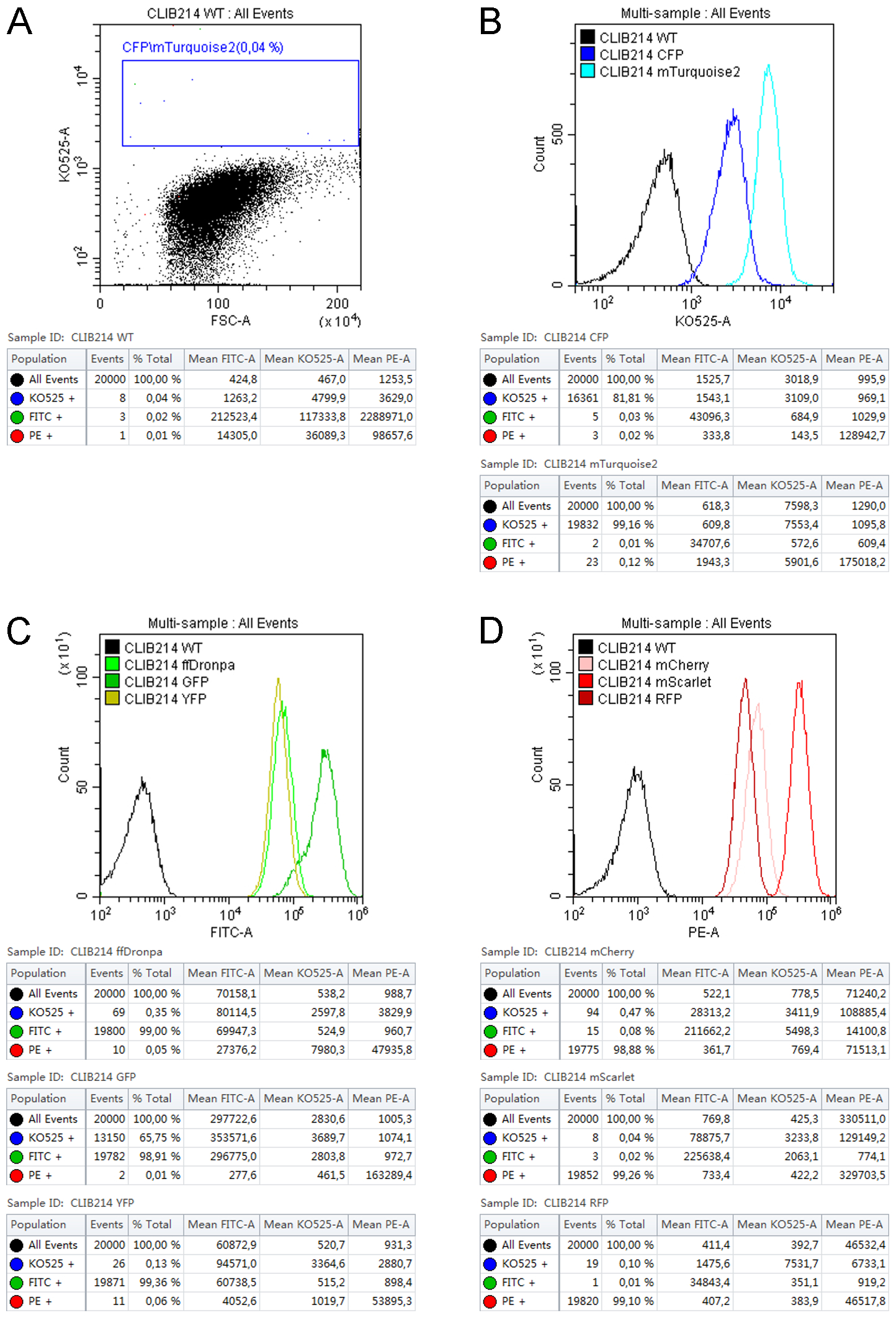

Supplement: S1 Fig — The parental strain was used to set up the proper gate (Panel A). The KO525 channel was applied for CFP and mTurquoise2 (Panel B), ffDronpa, GFP and YFP were detected in FITC channel (Panel C), and PE was used to detect the fluorescence of mCherry, mScarlet and RFP (Panel D). On the histograms the fluorescence intensities (x axis) of the FP expressing strains are compared to the one of parental strain. Statistical analysis of each mutant regarding the fluorescence in each channel is summarised below the given histograms. (TIF) [file pone.0312948.s001.tif]

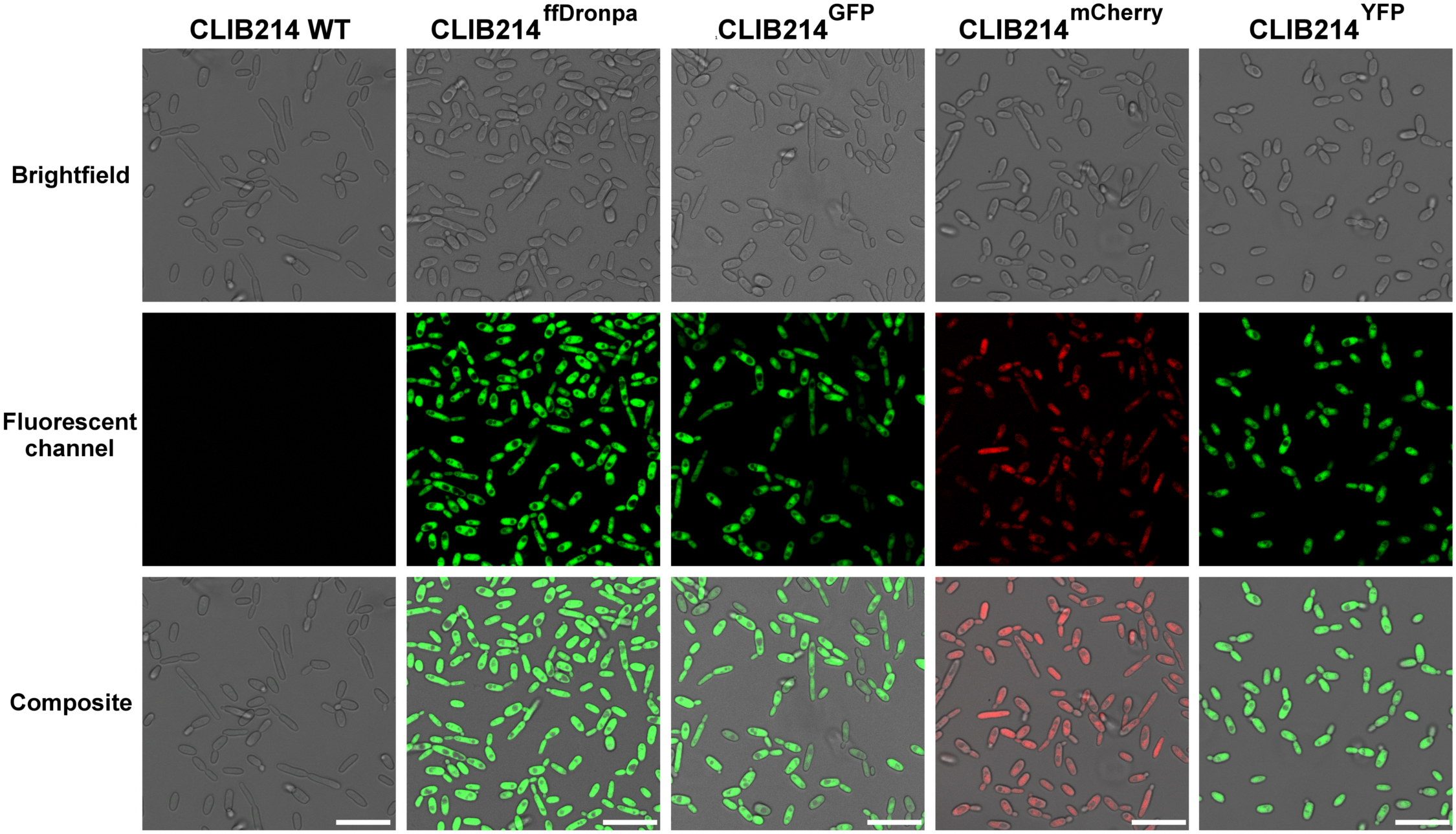

Supplement: S2 Fig — (TIF) [file pone.0312948.s002.tif]

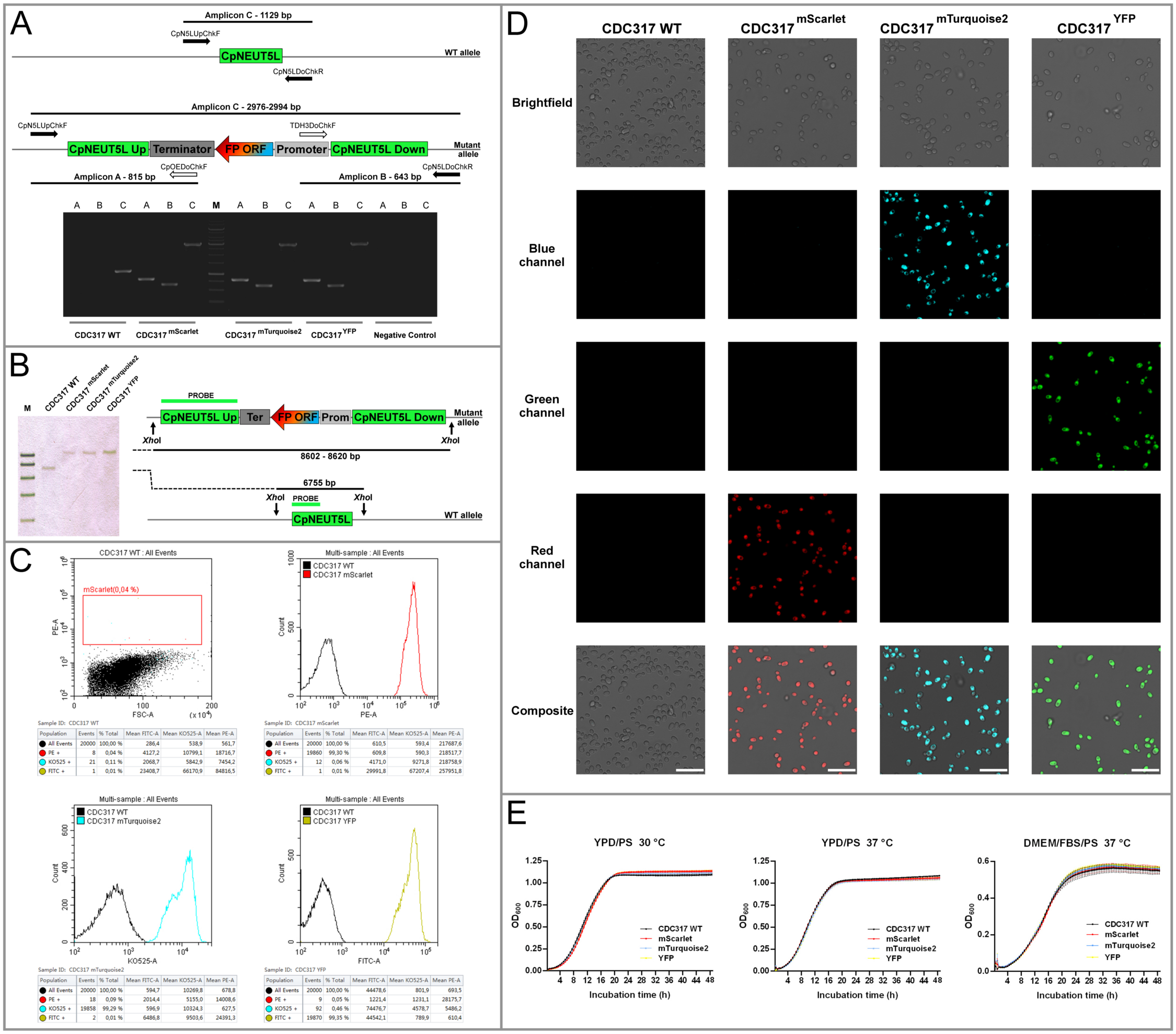

Supplement: S3 Fig — Panel A and Panel B show the validation by using PCR and southern-blot respectively. Panel C presents an example of the gating and the histograms using the parental strain as a reference in the given fluorescent channel. The statistics of the fluorescent signal is summarised below each histogram. Panel D summarises the microscopic images of the investigated strains. Panel E introduces the growth curves of the CDC317 isolate and its FP expressing strains grown in YPD/PS medium at 30 and 37 °C, and DMEM/FBS/PS medium at 37 °C. (TIF) [file pone.0312948.s003.tif]

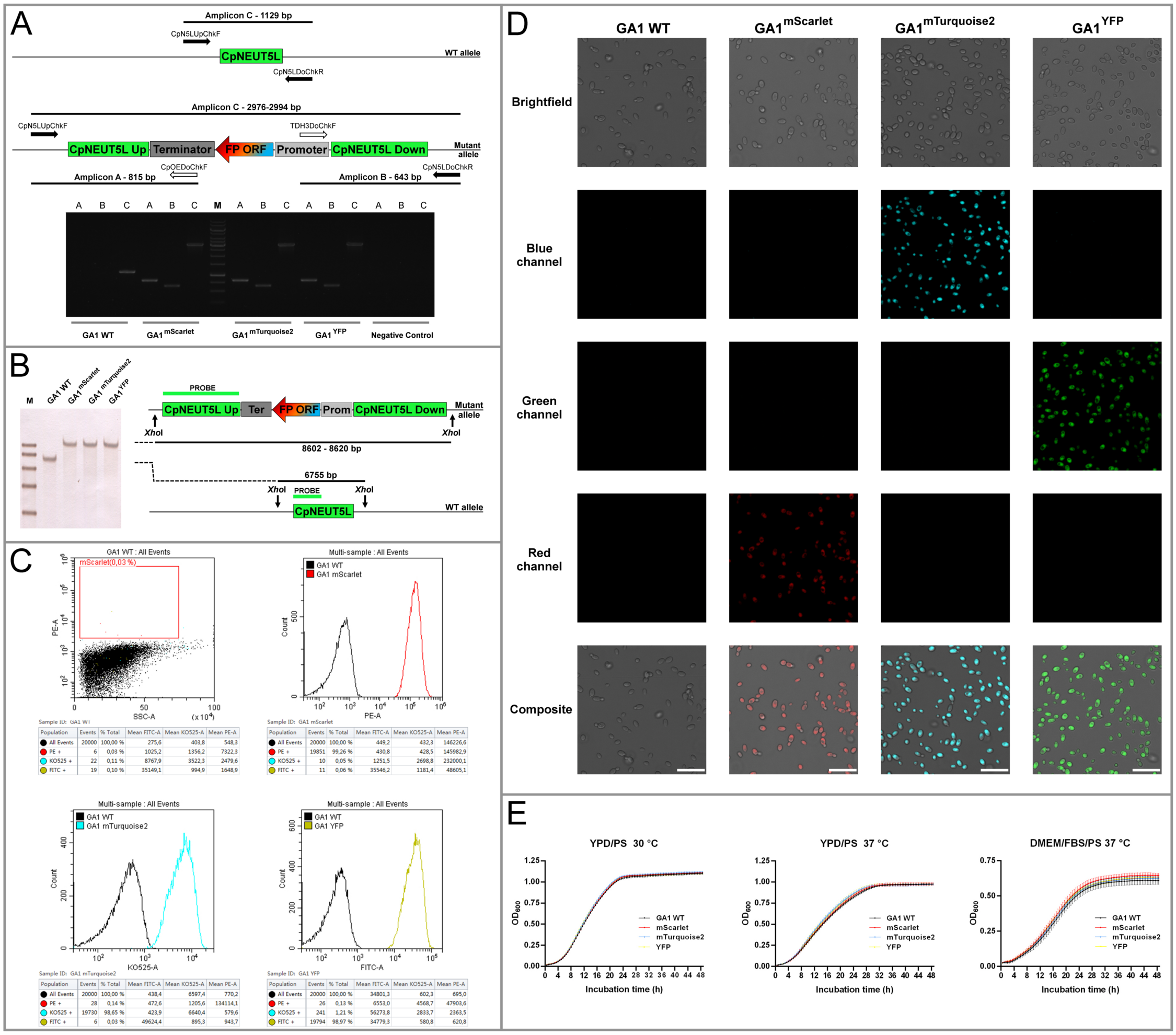

Supplement: S4 Fig — Panel A and Panel B show the validation by using PCR and southern-blot respectively. Panel C presents an example of the gating and the histograms using the parental strain as a reference in the given fluorescent channel. The statistics of the fluorescent signal is summarised below each histogram. Panel D summarises the microscopic images of the investigated strains. Panel E introduces the growth curves of the GA1 isolate and its FP expressing strains grown in YPD/PS medium at 30 and 37 °C, and DMEM/FBS/PS medium at 37 °C. (TIF) [file pone.0312948.s004.tif]

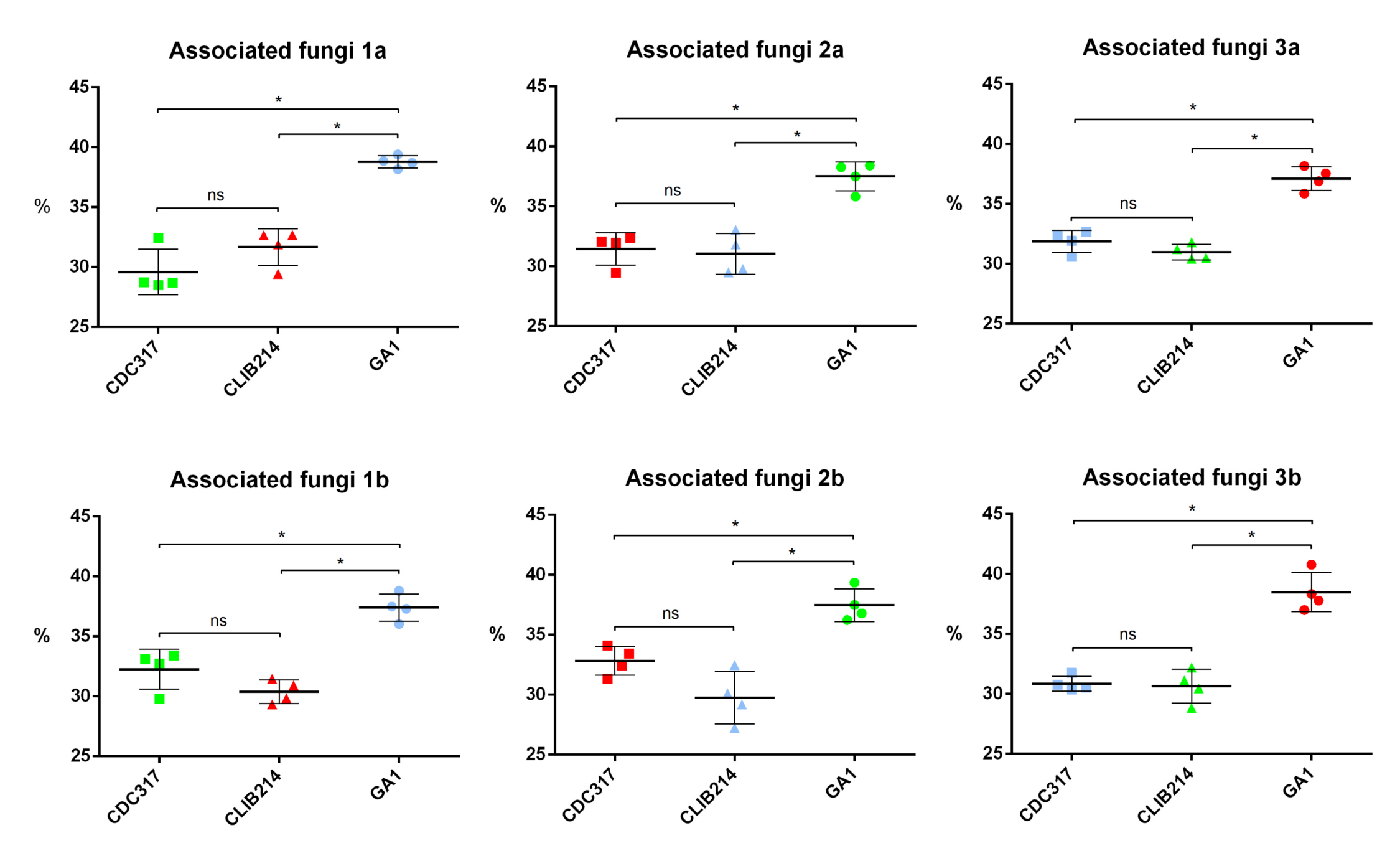

Supplement: S5 Fig — The J774.2 macrophage associated yeasts were counted and their ratio relative to the total number of macrophage associated yeasts was determined strain by strain. The shape of the signs indicate the strain (square: CDC317, triangle: CLIB214, circle: GA1), their colour refers to the applied FP in the given experiment (green: YFP, red: mScarlet, blue: mTurquoise2). The numbers in the titles illustrate the colour combination (columns), the “a” and “b” letters highlight the two repeats per colour-strain combination. One experiment was performed in biological duplicates, and two statistical parallels were applied per biological sample. Statistical analysis was performed according to Mann-Whitney test (ns: not significant, * : p < 0.05). (TIF) [file pone.0312948.s005.tif]

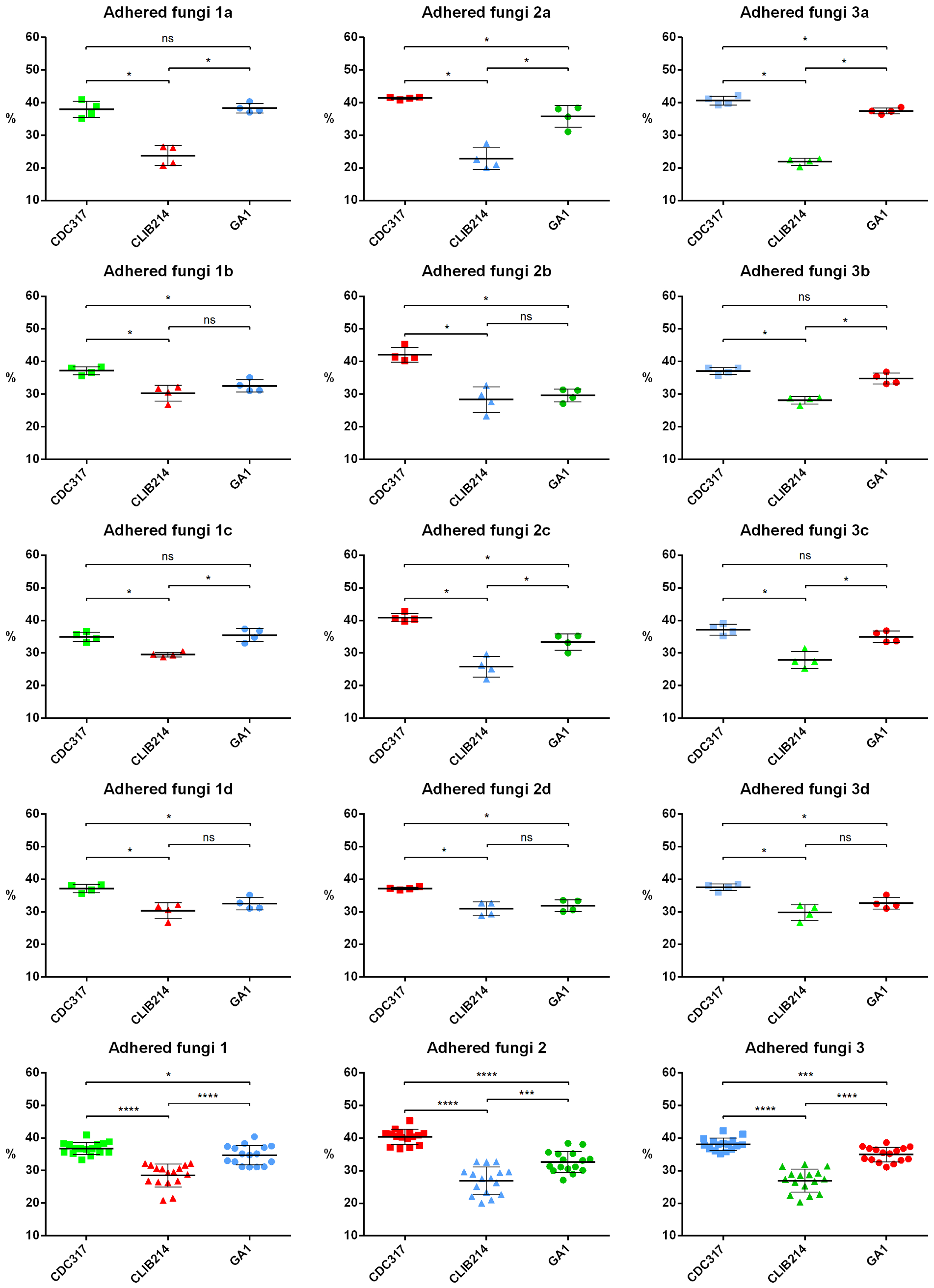

Supplement: S6 Fig — The yeasts adhered to silicone were counted, and their ratio relative to the total number of yeasts was determined strain by strain. The shape of the signs indicate the strain (square: CDC317, triangle: CLIB214, circle: GA1), their colour refers to the applied FP in the given experiment (green: YFP, red: mScarlet, blue: mTurquoise2). The numbers in the titles illustrate the colour combination (columns), the “a”, “b”, “c” and “d” letters indicate the four biological repeats per colour-strain combination. One experiment was performed in biological duplicates and two statistical parallels were applied per biological replicate. The results were summarised per colour-strain combination. Statistical analysis was performed according to Mann-Whitney test (ns: not significant, * : p < 0.05; ***: p < 0.001; ****: p < 0.0001). (TIF) [file pone.0312948.s006.tif]

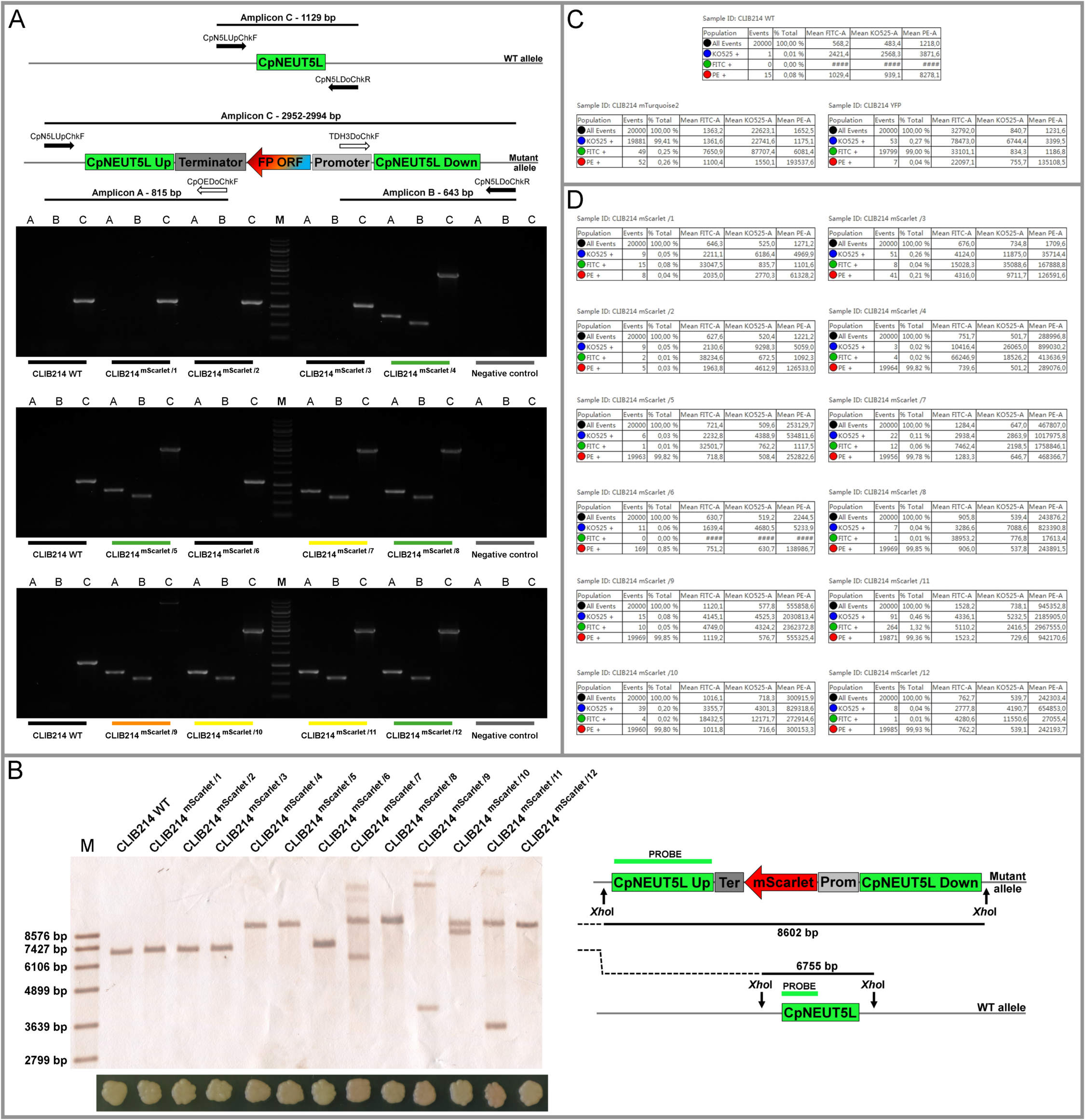

Supplement: S7 Fig — FP expressing derivatives of CLIB214 (1-12) were characterised by molecular methods and fluorescent imaging. Panel A and Panel B show the validation by using PCR and southern-blot respectively. The investigated transformants were pinned and cultivated on YPD/PS plate, and are presented under the filter. Panel C introduces the statistics of flow cytometry using the parental strain as a reference and the FP expressing CLIB214 mTurquoise2, and CLIB214 YFP for gating. Black line: WT/unaffected strain according to PCR and southern-blot, green line: PCR and southern-blot confirmed mutant, yellow line: mutant confirmed with PCR, but not with southern-blot, orange line: mutant not confirmed by PCR (Size of Amplicon “C” is not correct) and southern-blot. (TIF) [file pone.0312948.s007.tif]

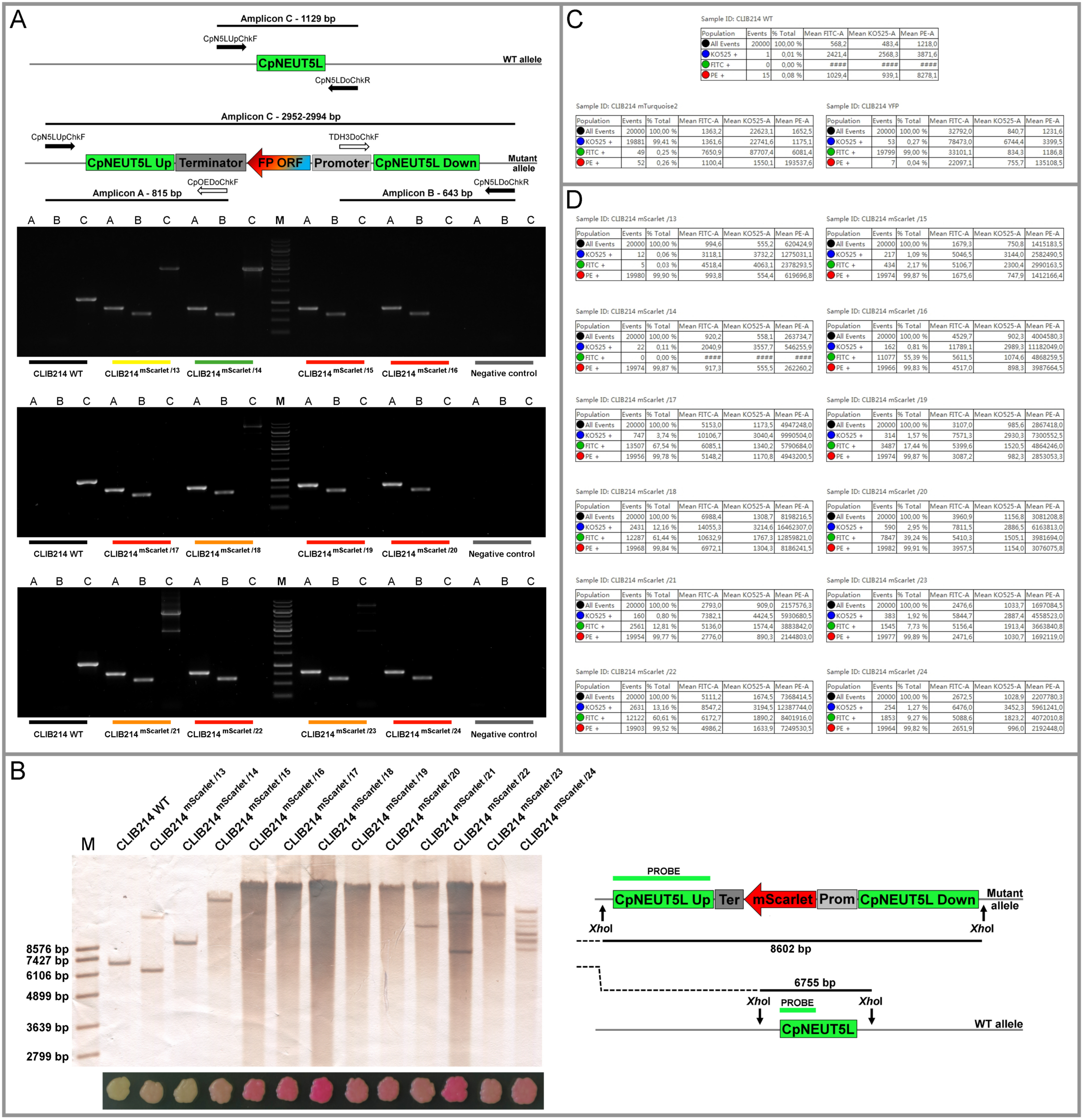

Supplement: S8 Fig — FP expressing derivatives of CLIB214 (13-24) were characterised by molecular methods and fluorescent imaging. Panel A and Panel B show the validation by using PCR and southern-blot respectively. The investigated transformants were pinned and cultivated on YPD/PS plate, and are presented under the filter. Panel C introduces the statistics of flow cytometry using the parental strain as a reference and the FP expressing CLIB214 mTurquoise2, and CLIB214 YFP for gating. Black line: WT/unaffected strain according to PCR and southern-blot, green line: PCR and southern-blot confirmed mutant, yellow line: mutant confirmed with PCR, but not with southern-blot, orange line: mutant not confirmed by PCR (Size of Amplicon “C” is not correct) and southern-blot, red line: mutant not confirmed by PCR (Amplicon “C” is missing) and southern-blot. (TIF) [file pone.0312948.s008.tif]
