# Supplementary material for: PCR-based CRISPR/Cas9 system for fluorescent tagging: A tool for studying Candida parapsilosis virulence
Source: PLoS One. 2025 Feb 24;20(2):e0312948. doi: 10.1371/journal.pone.0312948 (PMC12338950; doi:10.1371/journal.pone.0312948)
Supplement: S1 Appendix — (PDF) [file pone.0312948.s009.pdf]

Protocol for fluorescent labelling of *C. parapsilosis* cells with the PCR-based CRISPR/Cas9 method

DESCRIPTION

This genome editing approach utilises three plasmids for the CRISPR/Cas9 system and several additional ones for donor DNA (dDNA) generation. The Cas9 fragment can be released by digesting pTB101 with *Pme*I, that recombine *in vivo* with Fragment “C” generated by fusing the universal Fragment “A” and the specific Fragment “B” by PCR. To amplify Fragment “A” and Fragment “B” pTB120 and pTB121 are used as templates. The assembled cassette gets integrated into the *HIS1* locus of *C. parapsilosis* and confers resistance against nourseothricin. The expression of the single guide RNA is promoted by CPAR2_808670 (GAPDH) promoter and is protected by $tRNA^{ALA}$ and a hepatitis delta virus ribozyme at the 5’ and 3’ end, respectively. The Cas9 induces a double strand break at the site of the CpNEUT5L which is an intergenic region that can be modified without altering the fundamental stress tolerance and virulence properties of the fungus [1]. The dDNA encodes the desired fluorescent protein coding gene with regulatory sequences flanked by homologous arms of the target sequences lying up- and downstream from the double strand breakpoint. The dDNA can be amplified or released from a plasmid by digestion. The excision of the Cas9 cassette is initiated by incubating the resistant clone in the presence of maltose that leads to the expression of the site-specific recombinase flippase (FLP) which catalyses the recombination between the two flippase recognition sites (FRT) located at the ends of the construct. The sensitive mutant is compatible with upcoming application of the system however, transformants must be plated onto YNB-Drop out based media without histidine instead of complete YPD media to avoid the generation of histidine auxotrophs.

METHODS

$PEG_{8000}/MgCl_2$ method for DNA isolation

Note! By this method you can pellet any DNA longer than approximately 300 bp (typical primer dimers can be removed).

Materials

1x TE: prepared from 2M TRIS (pH=7.5) and 0.5 M EDTA (pH=8) with MilliQ water and autoclaved

$PEG_{8000}/MgCl_2$ solution: 30% (m/V) PEG_{8000} and 30 mM $MgCl_2$ dissolved in MilliQ water and autoclaved

Sterile molecular biology grade water

Procedure

1.) Transfer the PCR product to a new sterile microcentrifuge tube (leave ~ 2 µl in the tube)! (For example if the reaction mixture was 20 µl, take just 18 µl!)
2.) Add 3x volume sterile 1x TE (54 µl in this example)!
3.) Vortex for 2 seconds!
4.) Add half of the new volume $PEG_{8000}/MgCl_2$ solution (36 µl in this example)!
5.) Vortex the sample vigorously for one minute!
6.) Centrifuge at 17-18.000 g for 15 minutes!
7.) Carefully remove the supernatant!
8.) Centrifuge at 17-18.000 g for 10 seconds to collect leftover solution!
9.) Carefully remove as much residual supernatant as you can!
10.) Dissolve the pellet in sterile molecular biology grade water! The volume depends on the purpose and will be indicated later.

Determining the concentration of the DNA fragments

Note! Gel electrophoresis is suggested, but other methods e.g. nanodrop can also be used.

Materials

Agarose for gel electrophoresis
1x TAE
DNA loading dye
DNA molecular weight marker

Procedure

1.) Prepare an agarose gel (0.8-1% m/V) and load 1 µl of each diluted samples and assay them next to a DNA molecular weight marker! When checking an amplicon also include 1 µl of the unisolated PCR solution (This is what is left in the PCR tube at the first step of "**$PEG_{8000}/MgCl_2$ method for DNA isolation**")!
2.) Convert the ng/µl concentration to fmol/µl by using on-line calculators or the following formula: cc (fmol/µl) = [cc (ng/µl) * 1.000.000]/[660 * length of the product (bp)]

GENERAL NOTES

If you use different enzymes (DNA polymerase or restriction endonuclease) whose optimal conditions are distinct from the ones mentioned below, follow the instructions of the given protocol provided by the corresponding manufacturer.

dNTP mixture containing 1 mM of each nucleotide.

Dilute and prepare all solutions with sterile molecular biology grade water.

Primers are diluted to a concentration of 5 µM except for AHO1237 and AHO1232 that are 50 µM.

Generation of the fragments for transformation

Generation of Fragment „A"

Fragment „A" is the universal part of the Fragment „C". It encodes the downstream component of the NAT selection marker, the promoter for sgRNA expression and the $CptRNA^{ALA}$.

<u>Materials</u>
AHO1096 (5' -> 3'): GACGGCACGGCCACGCGTTTAAACCGCC (Tm:79.0 °C)
pTB120univR (5' -> 3'): TGGACGAGATAAGAATCGAACTCATG (Tm:65.3 °C)
pTB120 (1 ng/µl)

<u>Procedure</u>
1.) Set up the following reaction!

Reaction mixture (final volume: 50 µl)

- 10 µl ThermoFisher HF buffer for Phusion polymerase (5x)
- 10 µl dNTP mix
- 5 µl AHO1096 primer
- 5 µl pTB120univR primer
- 1 µl pTB120 plasmid template
- 0.5 µl ThermoFisher Phusion DNA polymerase (0.2 unit/µl)
- 18.5 µl Sterile molecular biology grade water

PCR thermal profile

98 °C, 30 sec
  98 °C, 10 sec
  65 °C, 30 sec } 35x
  72 °C, 45 sec
72 °C, 1:30 min
10 °C, ∞

Product size: 1478 bp

2.) Once the program is over, isolate the amplicon according to **„PEG_{8000}/$MgCl_2$ method for DNA isolation"**!
3.) Suspend the pellet in 50 µl sterile molecular biology grade water!
4.) Prepare a four step twofold dilution series to determine the concentration! (See „**Determining the concentration of the DNA fragments**" section!)
5.) Prepare a 40 fmol/µl working solution in sterile molecular biology grade water!

Generation of Fragment „B"

Fragment „B" is the varying, specific component of the Fragment „C". It encodes the gRNA scaffold, the hepatitis delta virus ribozyme, the terminator, the downstream flippase recognition site and the downstream homologous sequence of *CpHIS1* locus for targeted integration.

Materials
gRNA_CpN5L (5' -> 3'):
gagttcgattcttatctcgtcca**CTCTCGCTCCACTTCGGTCC**GTTTTAGAGCTAGAAATAGCAAG (Tm: 58.3 °C (specific) and 80.1 °C (complete))
AHO1097 (5' -> 3'): CCCGCCAGGCGCTGGGGTTTAAACACCG (Tm: 78.7 °C)
pTB121 (1 ng/µl)

Procedure
1.) Set up the following reaction!
Note! When calculating the annealing temperature, use only the sequence specific to the template alone (indicated in NOT bold, capital letters)!

Reaction mixture (final volume: 20 µl)
- 4 µl ThermoFisher HF buffer for Phusion polymerase (5x)
- 4 µl dNTP mix
- 2 µl gRNA_CpN5L primer (5 µM)
- 2 µl AHO1097 primer (5 µM)
- 1 µl pTB121 plasmid template
- 0.2 µl ThermoFisher Phusion DNA polymerase (0.2 unit/µl)
- 6.8 µl Sterile molecular biology grade water

PCR thermal profile
- 98 °C, 30 sec
  - 98 °C, 10 sec
  - 60 °C, 30 sec -1 °C/cycle } 5x
  - 72 °C, 25 sec
  - 98 °C, 10 sec
  - 72 °C, 50 sec } 30x
- 72 °C, 50 sec
- 10 °C, ∞

Product size: 845 bp

2.) Isolate the amplicon according to **„PEG$_{8000}$/MgCl$_2$ method for DNA isolation"**!
3.) Suspend the pellet in 20 µl sterile molecular biology grade water!
4.) Prepare a four step twofold dilution series to determine the concentration! (See „**Determining the concentration of the DNA fragments**" section!)
5.) Set the concentration to 40 fmol/µl by using sterile molecular biology grade water!

Generation of transformation ready Fragment „C"
This process utilizes two separate reactions, the first without the primers.
Note! According to our experiences one transformation requires Fragment „C" amplicons from three individual PCRs of 50 µl, so scale accordingly.

Materials
AHO1232 (5' -> 3'): AGAATGCCTATTGACTTCAAAGGTG (Tm: 64.4 °C)
AHO1237 (5' -> 3'): AGGTGATGCTGAAGCTATTGAAG (Tm: 63.8 °C)
Universal Fragment „A" (40 fmol/µl)
Specific Fragment „B" (40 fmol/µl)

Procedure

1.) Set up the following reaction!

Reaction mixture 1 (final volume: 49 µl)

- 10 µl ThermoFisher HF buffer for Phusion polymerase (5x)
- 10 µl dNTP
- 1 µl Universal Fragment „A”
- 1 µl Specific Fragment „B”
- 0.5 µl ThermoFisher Phusion DNA polymerase (2 unit/µl)
- 26.5 µl Sterile molecular biology grade water

PCR thermal profile 1

- 98 °C, 30 sec
  - 98 °C, 10 sec
  - 63 °C, 30 sec } 5x
  - 72 °C, 1:00 min
- 10 °C, ∞

2.) While Reaction 1 is running, prepare a working solution of AHO1237 and AHO1232 primer mix by setting up the following mixture (for one sample)!

- 0.25 µl AHO1237 (100 µM)
- 0.25 µl AHO1232 (100 µM)
- 0.5 µl Sterile molecular biology grade water

Note! Always calculate for one additional sample! (For instance, if you have three tubes of PCR running, prepare a primer mixture for four!)
If you plan to use this technique regularly, it is advised to prepare a larger volume of the working primer solution to avoid thawing-freezing cycles of the stocks.

3.) Set up Reaction mixture 2 by adding 1 µl of AHO1232/AHO1237 primer mix to the Reaction mixture 1

PCR thermal profile 2

- 98 °C, 30 sec
  - 98 °C, 10 sec
  - 63 °C, 30 sec } 35x
  - 72 °C, 1:10 min
- 72 °C, 2:00 min
- 10 °C, ∞

Product size: 2220 bp

4.) Once the amplification is over, isolate the product as described at **„$PEG_{8000}/MgCl_2$ method for DNA isolation”** section!
Note! Put **not more than** 200 µl PCR mixture into a single 1.5 ml tube!
5.) Suspend the pellet in 25 µl sterile molecular biology grade water!
6.) Prepare 10x, 20x and 40x dilutions!

7.) Determine the concentration of the amplicon according to „**Determining the concentration of the DNA fragments**” section!

Generation of transformation ready Cas9 fragment

Materials
LB liquid media (1% m/V sodium chloride, 1% m/V tryptone, 0.5% m/V yeast extract) supplemented with ampicillin in a final concentration of 50 µg/ml
3 M Na-acetate (pH=5.8)
Isopropanol
70% (V/V) ethanol

Procedure
1.) Propagate the pTB101 in a large scale (midi or maxiprep) by using a commercially available plasmid isolation kit!
Note! The pTB101 carries AMPICILLIN resistance marker.
2.) Prepare a dilution series (5x, 10x, 20x) of the isolated plasmid and digest it with *Eco*RI!
3.) Determine the concentration of the plasmid as described in „**Determining the concentration of the DNA fragments**” section! (*Eco*RI digested pTB101 should have the following bands: 3457 bp + 2515 bp + 1908 bp + 1700 bp + 1178 bp + 804 bp)
4.) Set up an overnight plasmid digestion as follows:
    50 µl ThermoFisher Buffer Blue (10x)
    12 µg pTB101
    4 µl ThermoFisher *Mss*I (*Pme*I) 5u/µl
    Sterile molecular biology grade water to 500 µl
5.) Gently vortex and spin down!
6.) Incubate overnight at 37 °C!
7.) Next morning add 50 µl (1/10 volume) of 3 M Na-acetate (pH=5.8) to the digestion reaction and mix gently!
8.) Add 550 µl (equal volume) isopropanol and vortex the sample vigorously for 5 seconds!
9.) Keep the sample in a -20 °C freezer for at least 20 minutes!
10.) Centrifuge the sample for 10 minutes at 17.000-18.000 g!
11.) Remove the supernatant!
12.) Pipette 600 µl of 70% (V/V) ethanol onto the pellet!
13.) Centrifuge the sample for 10 minutes at 17.000-18.000 g!
15.) Remove the supernatant and let the pellet dry on the bench!
16.) Suspend the pellet in 15 µl sterile molecular biology grade water! (If you performed the digestion in more tubes, then scale this volume up accordingly and wash the pellets together to have one single tube at the end!
17.) Prepare 5x, 10x, 20x dilutions of the digested plasmid
18.) Determine the concentration of the Cas9 fragment according to „**Determining the concentration of the DNA fragments**” section! The *Pme*I digestion yields a 9336 bp and a 2226 bp fragment. The larger is the Cas9 fragment!

Generation of the FP coding dDNA

The currently available plasmid collection is suitable to fluorescently tag *C. parapsilosis* with CFP, ffDronpa, GFP, mCherry, mScarlet, mTurquoise2, RFP and YFP. However, CFP and RFP are avoided due to their weak fluorescent signal. The transcription is regulated by the TDH3 promoter of *C. albicans* and the URA3 terminator of *S. cerevisiae*. In addition, this sequence is flanked by the homologous arms of the targeted sequence, NEUT5L of *C. parapsilosis*. The dDNAs can be generated by PCR amplification or they can be released by plasmid digestion.
Note! We found that one transformation requires three individual PCRs of 50 µl, so scale accordingly.

Materials
LB liquid media (1% m/V sodium chloride, 1% m/V tryptone, 0.5% m/V yeast extract) supplemented with kanamycin in a final concentration of 50 µg/ml

Procedure
1.) Propagate the required pNRVL-N5L-FP. If the dDNA will be gained by plasmid digestion a larger amount of pNRVL-N5L-FP is required (midiprep or maxiprep is needed). If the dDNA will be amplified by PCR a small amount of plasmid is sufficient (miniprep is enough).
Note! The pNRVL-N5L plasmids carry KANAMYCIN resistance marker.
2.) Prepare a dilution series (5x, 10x, 20x) of the midi- or maxipreps or use the undiluted miniprep and digest it with the appropriate restriction endonuclease!
3.) Determine the concentration of the plasmid as described in „**Determining the concentration of the DNA fragments**” section!

dDNA generation by PCR

FP_CpN5L_dDNA_F (5' -> 3'): AACCTCATCTCAAGGCGC (Tm: 62.8 °C)
FP_CpN5L_dDNA_R (5' -> 3'): ACACAAAAATACATGATTGCGTC (Tm: 61.5 °C)
pNRVL-N5L-FP (1 ng/µl)

Procedure
1.) Set up the following reaction!

Reaction mixture (final volume: 50 µl)
- 10 µl ThermoFisher HF buffer for Phusion polymerase (5x)
- 10 µl dNTP (1-1 mM each)
- 5 µl FP_CpN5L_dDNA_F (5 µM)
- 5 µl FP_CpN5L_dDNA_R (5 µM)
- 1 µl pNRVL-N5L-FP (1 ng/µl)
- 0.5 µl ThermoFisher Phusion DNA polymerase (2 unit/µl)
- 18.5 µl Sterile molecular biology grade water

PCR thermal profile
- 98 °C, 30 sec
  - 98 °C, 10 sec
  - 60 °C, 30 sec } 35x
  - 72 °C, 1:30 min
- 72 °C, 3:00 min
- 10 °C, ∞

Product size: CFP: 2649 bp, ffDronpa: 2607 bp, GFP: 2649 bp, mCherry: 2643 bp, mScarlet: 2631 bp, mTurquoise2: 2649 bp, RFP: 2610 bp, YFP: 2649 bp

2.) Once the amplification is over, isolate the amplicon by using the **$PEG_{8000}/MgCl_2$ method** (leave 2 µl reaction mixture in one of the PCR tubes)
3.) Suspend the pellet in 20 µl sterile molecular biology grade water (if 3x50 µl PCR product was purified)!
If more was set up, scale the volume up accordingly, and wash the samples in one single tube!
4.) Prepare 10x, 20x and 40x dilutions and determine the concentration as described in „**Determining the concentration**" section!

dDNA generation by plasmid digestion

The dDNA can be released by *Stu*I digestion from the dedicated pNRVL-N5L-FP plasmid. The reaction yields two fragments: the plasmid backbone (2338 bp) and the dDNA fragment (2613 – 2655 bp depending of the FP). Calculate one digestion reaction per transformation.

Materials
3 M Na-acetate (pH=5.8)
Isopropanol
70% (V/V) ethanol

Procedure
1.) Set up an overnight plasmid digestion as follows:

Reaction mixture (final volume 500 µl)
50 µl ThermoFisher Buffer Blue (10x)
12 µg pNRVL-N5L-FP
2 µl ThermoFisher *Stu*I 10u/µl
Sterile molecular biology grade water to 500 µl

2.) Gently vortex and spin down!
3.) Incubate overnight at 37 °C!
(Alternatively, if you have the enzyme from a different source, you can adjust these parameters according to the manufacturer's protocol.)
4.) The next morning add 50 µl (1/10 volume) of 3 M Na-acetate (pH=5.8) to the digestion reaction and mix gently!
5.) Add 550 µl (equal volume) isopropanol and vortex the sample(s) vigorously for 5 seconds!
6.) Place the sample(s) in a -20 °C freezer for at least 20 minutes!
7.) Centrifuge the sample(s) for 10 minutes at 17.000-18.000 g!
8.) Remove the supernatant!
9.) Pipette 600 µl of 70% (V/V) ethanol onto the pellet(s)!
10.) Centrifuge the sample(s) for 10 minutes at 17.000-18.000 g!
11.) Remove the supernatant and let the pellet(s) dry on the bench!
12.) Suspend the pellet(s) in 15 µl sterile molecular biology grade water! (If you performed the digestion in more tubes, then scale this volume up accordingly and wash the pellets into one single tube!
13.) Prepare 5x, 10x, 20x dilutions and determine the concentration of the Cas9 fragment according to „**Determining the concentration of the DNA fragments**" section!

Transformation of *C. parapsilosis* (taken from Németh et al., 2021)

Materials

YPD liquid media for cultivation: 1% (m/V) glucose, 1% (m/V) peptone, 0.5% (m/V) yeast extract, autoclaved.

YPD/PS: YPD liquid media supplemented with penicillin-streptomycin (PS) at a final concentration of 100 unit/ml after autoclave.

YPD/PS plate: 1% (m/V) glucose, 1% (m/V) peptone, 0.5% (m/V) yeast extract + 2% (m/V) agar, autoclave and add penicillin-streptomycin (PS) at a final concentration of 100 unit/ml!

YPD/PS/NTC^{2} and YPD/PS/NTC^{100} plates: YPD/PS plates supplemented with nourseothricin (NTC) after autoclave in a final concentration of 2 or 100 µg/ml (YPD/PS/NTC^{2} and YPD/PS/NTC^{100} respectively)!

Note! When cumulative mutations are generated YNB+Dropout (YNB+DO) solid mediums instead of YPD mediums have to be used from the second round of transformation to provide selective pressure to avoid the emergence of histidine auxotrophy! This will be highlighted later in the protocol by a * sign as a reminder.

10x Drop out (DO) medium (1 litre):

L-Arginine 200 mg
L-Isoleucine 300 mg
L-Leucine100 mg
L-Lysine 300 mg
L-Methionine 200 mg
L-Phenylalanine 500 mg
L-Threonine 2000 mg
L-Tyrosine 300 mg
L-Tryptophan 2000 mg
L-Valine 1500 mg
Adenine 2000 mg
Uracil 200 mg

YNB+DO/PS plates: 0.19% (m/V) Yeast nitrogen base, 2% (m/V) glucose, 2% (m/V) agar autoclaved and then supplemented with penicillin-streptomycin (PS) at a final concentration of 100 unit/ml!

YNB+DO/PS/NTC^{2} and YNB+DO/PS/NTC^{100} plates: YNB+DO/PS plates supplemented with NTC after autoclave in a final concentration of 2 or 100 µg/ml (YPD/PS/NTC^{2} and YPD/PS/NTC^{100} respectively)!

Sterile MilliQ water
0.5 M EDTA (pH=7.5), sterile
2 M tris(hydroxymethyl)aminomethane (pH=8), sterile

On the day of the transformation (during the cultivation period) prepare **freshly**:

- 10x TE: combine 20 µl 0.5 M EDTA (pH=7.5) and 50 µl 2 M tris(hydroxymethyl)aminomethane (pH=8) stocks solutions in 930 µl MilliQ distilled water and autoclave! Scale this volume up, if necessary!
- 1 M lithium-acetate: dissolve 102 mg Lithium-acetate in 950 ml MilliQ distilled water and autoclave! Scale this volume up, if necessary!

- 55% (m/V) PEG_{3350}: weigh first the PEG_{3350} powder in a 15 or 50 ml conical tube then set the volume with sterile MilliQ water! Dissolve it completely and then autoclave!

Use these sterile 10x TE, 1M lithium-acetate and PEG_{3350} as stocks to prepare:

- 1x TELioAc (1/10 volume of 1 M lithium-acetate, 1/10 volume of 10x TE and 8/10 volume of sterile MilliQ water)
- DMSO/$PLATE_{3350}$ solution (1/10 volume of 1 M Lithium-acetate, 1/10 volume of 10x TE, 8/10 volume of 55% (m/V) PEG_{3350}. Then add sterile DMSO to the PLATE solution in a ratio of 1:10 (DMSO:PLATE) and protect it from light later on.
- Place (number of transformation + 1) x 10 µl salmon sperm in boiling water in a microcentrifuge tube for 10 minutes and then cool it down rapidly on wet ice (ssDNA) and keep it on ice.

Perform experiment at room temperature unless otherwise stated!

Procedure

Day one

1.) Inoculate the *C. parapsilosis* strain in 5 ml YPD and incubate overnight at 30 °C (~150 rpm)!

Day two

2.) Adjust OD_{600} to 0.05 in YPD in a 250 or 500 ml glass flask (the volume of the suspension should not exceed the 1/3 of the total volume of the flask) and incubate at 30 °C (~150 rpm)
Note! When using CLIB214 the cultivation will last about 7.5 hours
3.) Prepare the 1x TELioAc, DMSO:PLATE and ssDNA as indicated above
4.) When OD_{600} = 1.34 ± 0.05, transfer 2.75 ml suspension/transformation into a 15 or 50 ml falcon tube (depends on the volume)! (Always calculate for one more transformation! For instance for three transformations [(3+1 additional) x 2.75 ml =] 11 ml suspension should be taken.)
5.) Centrifuge at ~2000 g, 5 minutes!
6.) Remove the supernatant and suspend the cells in half a volume of sterile MilliQ water
(If you took for instance 11 ml of suspension, use ~5.5 ml of water)
7.) Centrifuge for 5 minutes, ~2000 g, during this time
8.) Set up the transformation mixture(s) in 1.5 ml microcentrifuge tube(s) on ice as follows:

500 fmol Cas9 fragment
2500 fmol Fragment „C”
2500 fmol dDNA
10 µl ssDNA (boiled and ice cooled)

Mix contents, then spin the tubes down rapidly in a bench top centrifuge and keep them on ice! This amount of transforming DNA should yield 20-30 colonies.
9.) Remove the supernatant and suspend the cells in 1 ml 1x TELioAc!
10.) Transfer the suspension to a sterile 1.5 ml microcentrifuge tube and centrifuge in a bench top centrifuge ~17-18,000 g, 2 minutes!
11.) Remove supernatant and suspend the pellet in 50 µl 1x TELioAc/transformation! (For instance if You have three transformations suspend the pellet in (3+1 additional) x 50 µl = 200 µl)
12. Add 50 µl of this suspension to the reaction mixture(s) from Step 8) and mix gently!

13.) Add 770 µl freshly prepared DMSO/PLATE$_{3350}$ solution immediately, then turn the tubes upside down and flick them gently three times to mix contents!
14.) Incubate for ~15 hours (practically until the next morning) at 30 °C overnight (static)!

Day three
15.) Perform heat shock by placing the samples into a water bath (44 °C for 15 minutes)!
16.) Centrifuge 17,000 g, 2 minutes (benchtop centrifuge)!
17.) Carefully remove as much supernatant as possible!
18.) Add 950 µl of YPD without disturbing the pellet, then turn the tubes upside down and flick them gently three times to bring remaining DMSO/PLATE$_{3350}$ in a solution with YPD!
19.) Centrifuge at 17,000 g, for 2 minutes (benchtop centrifuge)!
20.) Remove as much supernatant as possible and suspend the pelleted cells gently in 300 µl YPD!
21.) Incubate the tubes at 30 °C with shaking (~150 rpm) for 5 hours!
22.) Centrifuge at 2,400 g for 5 minutes (benchtop centrifuge)!
23.) Remove 200 µl of the supernatant!
24.) Suspend the pellet in the rest of the supernatant and plate the suspension onto YPD/PS/NTC^{100} plate (or onto YNB+DO/PS/NTC^{100} plate, if the parental strain has already been edited with this system)*!
25.) Incubate at 30 °C for 2-3 days!

Validation of the transformants by colony PCR (based on Holland et al., 2014)

According to our experience, we advise to collect 12 NTC resistant transformants. The integration of the dDNA should be verified by three PCRs. Two for the region of the up (Amplicon A)- and downstream (Amplicon B) parts and one verifying the complete locus (Amplicon C). The use of DreamTaq or other DNA polymerase without proofreading activity is suggested to reduce costs.

Materials
Lysis buffer: 1% (m/V) sodium-dodecyl-sulphate, 100 mM Lithium-acetate dissolved in distilled water
96% (V/V) ethanol
70% (V/V) ethanol
Sterile molecular biology grade water
YPD/PS/NTC^{2} or YNB+DO/PS/NTC^{2} plate
YPD/PS/NTC^{100} or YNB+DO/PS/NTC^{100} plate
YNB+maltose liquid media: 0.19% (m/V) Yeast nitrogen base, 2% (m/V) maltose, dissolved in distilled water and autoclaved
Sterile 1x PBS

Procedure
Day one
1.) Label a YPD/PS/NTC^{100} plate* as a master plate to keep NTC resistant clones!
2.) Take one 1.5 ml and one 2 ml sterile microcentrifuge tubes per colony PCR!
3.) Pipette 15 µl sterile molecular biology grade water into the 2 ml microcentrifuge tube!
4.) Pipette 90 µl Lysis buffer into the 1.5 ml microcentrifuge tube!
5.) Set the pipette to 10 µl, and gently take a tiny sample from the transformant colony!
6.) Suspend the cells into the 15 µl water and take 10 µl suspension!
7.) Gently touch the surface of the YPD/PS/NTC^{100} plate* to pin the cells onto the media!

8.) Suspend the rest of the suspension into the 90 µl Lysis buffer in the 1.5 ml microcentrifuge tube!
9.) Perform Steps 5.) - 8.) with the remaining colonies!
10.) Incubate the samples in the lysis buffer at 70 °C for 5 minutes!
11.) Place them on ice for five minutes!
12.) Add 300 µl (3x volume) 96% (V/V) ethanol!
13.) Mix vigorously for one minute with a vortex!
14.) Centrifuge the samples in a benchtop centrifuge for 5 minutes (~17.000-18.000 g)!
15.) Remove the supernatant!
16.) Add 200 µl 70% (V/V) ethanol!
17.) Centrifuge the pellet again for 3 minutes (~17.000-18.000 g)!
18.) Remove the supernatant!
19.) Let the pellet dry on the bench!
20.) Pipette 50 µl sterile molecular biology grade water onto the pellet and vortex for 3 minutes to suspend!
21.) Set up the PCRs (use wild-type DNA control and one without a template as a negative control)

Reaction mixture (final volume: 20 µl)

- 2 µl DreamTaq buffer (10x)
- 4 µl dNTP (1-1 mM each)
- 1 µl forward primer (5 µM)
- 1 µl reverse primer (5 µM)
- 1 µl template DNA (from the DNA isolation above)
- 0.2 µl DreamTaq polymerase (5u/µl)
- 10.8 µl sterile molecular biology grade water

For „Amplicon A” use primers:

CpN5LUpChkF (5’ -> 3’): CCTATTTCGCATCATTGCAGTTC (Tm: 58.8 °C)
CpOEDoChkF (5’ -> 3’): ACCCGGGAATCTCGGTCG (Tm: 62.8 °C)
Product size: 815 bp

For „Amplicon B” use primers:
TDH3DoChkF (5’ -> 3’): TTTCGGAATTGAACCACGCG (Tm: 58.9 °C)
CpN5LDoChkR (5’ -> 3’): TGTACCAATCAGGGTTAGTGACC (Tm: 61.1 °C)
Product size: 643 bp

PCR thermal profile for Fragment „A” and „B”

94 °C, 1 min
- 94 °C, 20 sec
- 58 °C, 25 sec } 35x
- 72 °C 1 min

72 °C, 2 min
10 °C, ∞

For „Amplicon C” use primers:

CpN5LUpChkF (5’ -> 3’): CCTATTTCGCATCATTGCAGTTC (Tm: 58.8 °C)
CpN5LDoChkR (5’ -> 3’): TGTACCAATCAGGGTTAGTGACC (Tm: 61.1 °C)

Product size: 1129 bp (WT), 2952-2994 bp (mutant carrying the FP ORF)

PCR thermal profile for Fragment „C"

94 °C, 1 min

94 °C, 20 sec
58 °C, 25 sec } 35x
72 °C 3 min

72 °C, 6 min

10 °C, ∞

22.) Assay the amplicons on an agarose gel (0.8-1% m/V)!
23.) Pick the 2 ml microcentrifuge tubes with the 5 µl suspension of the PCR verified NTC resistant clones and add 300 µl YNB-maltose liquid media! (Tubes with unverified clones can be thrown in away and must be handled as a biohazardous material!)
24.) Incubate the samples in an orbital shaker at 30 °C (~150 rpm) overnight!

Day two
27.) Take the tubes with verified clones!
28.) Prepare a tenfold dilution of the suspension in sterile 1xPBS, and determine the cell concentration by using a hemocytometer!
29.) Set the cell number to 3,000 cells/ml!
30.) Spread 100 µl suspension onto YPD/PS/NTC^{2} (or YNB+DO/PS/NTC^{2})* plate!
31.) Place the plate to 30 °C and incubate for two days!

Day four
32.) Take a YPD/PS (or YNB+DO/PS)* plate and label six sectors on its back!
33.) Carefully pick three small colonies from the YPD/PS/NTC^{2} (or YNB+DO/PS/NTC^{2}) plate with a sterile toothpick and scratch them onto a YPD/PS (or a YNB+DO/PS)* plate in a zig-zag line to gain single cell colonies!
34.) Incubate the plate at 30 °C for one day!

Day five
35.) Label a YPD/PS (or a YNB+DO/PS)* master plate!
36.) Pick a single cell colony with a sterile toothpick from the zig-zag line carefully and transfer it to the master plate!
37.) Incubate the plate for one day at 30 °C!

Day six
38.) To test the excision of the cassette, label a YPD/PS and a YPD/PS/NTC^{100} plate in the same pattern (similarly to a master plate)!
39.) According to the number of the strains to be tested label as many plus three 1.5 sterile microcentrifuge tubes and pipette 100 µl sterile 1x PBS into each of them! (Plus three is for the parental strain, an NTC resistant control and a 1x PBS control.)
40.) Set the pipette to 4 µl and take a tiny inoculum from the colonies on the master plate from Step 37)!
41.) Suspend the cells in the sterile 1x PBS!
42.) Pipette 4 µl suspension onto the YPD/PS and the YPD/PS/NTC^{100} plate!
43.) Incubate the plates for two days at 30 °C!

The clones not being able to grow in the presence of the NTC are actual transformants and are compatible with the subsequent application of this system.

Before using the transformants in any subsequent transformation or experiment a verification with PCR (once again), southern-blot and recording the growth curve in complete media are strongly recommended!

REFERENCES

1. Nemeth T, Papp C, Vagvolgyi C, Chakraborty T, Gacser A. Identification and Characterization of a Neutral Locus for Knock-in Purposes in C. parapsilosis. Front Microbiol. 2020;11: 1194. doi:10.3389/fmicb.2020.01194
2. Németh T, D Nosanchuk J, Vagvolgyi C, Gacser A. Enhancing the chemical transformation of Candida parapsilosis. Virulence. 2021;12: 937–950. doi:10.1080/21505594.2021.1893008
3. Holland LM, Schröder MS, Turner SA, Taff H, Andes D, Grózer Z, et al. Comparative phenotypic analysis of the major fungal pathogens Candida parapsilosis and Candida albicans. PLoS Pathog. 2014;10: e1004365. doi:10.1371/journal.ppat.1004365
